# Dominant and genome-wide formation of DNA:RNA hybrid G-quadruplexes in living yeast cells

**DOI:** 10.1073/pnas.2401099121

**Published:** 2024-10-23

**Authors:** Chen-xia Ren, Rui-fang Duan, Jia Wang, Yu-hua Hao, Zheng Tan

**Affiliations:** ^a^Shanxi Key Laboratory of Aging Mechanism Research and Translational Applications, Center for Healthy Aging, Central Laboratory, Changzhi Medical College, Changzhi, Shanxi 046000, People’s Republic of China; ^b^State Key Laboratory of Membrane Biology, Institute of Zoology, Chinese Academy of Sciences, Beijing 100101, People’s Republic of China

**Keywords:** DNA:RNA hybrid G-quadruplex, Okazaki fragment, genome, replication, transcription

## Abstract

Guanine-rich DNA forms G-quadruplexes (G4s) that play a critical role in essential cellular processes. Previous studies have mostly focused on intramolecular G4s composed of four consecutive guanine tracts (G-tracts) from a single strand. However, this structural form has not been strictly confirmed in the genome of living eukaryotic cells. Here, we report the formation of hybrid G4s (hG4s), consisting of G-tracts from both DNA and RNA, in the genome of living yeast cells. Analysis of Okazaki fragment syntheses and two other independent G4-specific detections reveal that hG4s can efficiently form with as few as a single DNA guanine–guanine (GG) tract due to the participation of G-tracts from RNA. This finding increases the number of potential G4-forming sites in the yeast genome from 38 to 587,694, a more than 15,000-fold increase. Interestingly, hG4s readily form and even dominate at G4 sites that are theoretically capable of forming the intramolecular DNA G4s (dG4s) by themselves. Compared to dG4s, hG4s exhibit broader kinetics, higher prevalence, and greater structural diversity and stability. Most importantly, hG4 formation is tightly coupled to transcription through the involvement of RNA, allowing it to function in a transcription-dependent manner. Overall, our study establishes hG4s as the overwhelmingly dominant G4 species in the yeast genome and emphasizes a renewal of the current perception of the structural form, formation mechanism, prevalence, and functional role of G4s in eukaryotic genomes. It also establishes a sensitive and currently the only method for detecting the structural form of G4s in living cells.

## Significance

Our work reveals the genome-wide formation of a pervasively dominant but virtually unnoticed type of G-quadruplexes (G4s), i.e., DNA:RNA hybrid G-quadruplexes (hG4s), in the genome of living eukaryotic cells. The involvement of RNA confers two important features to hG4s: 1) the ability of genomic DNA to form G4s with as few as one G-tract instead of four, resulting in the complete dominance of hG4s over canonical monomeric DNA G4s (dG4s) and their ubiquitous presence in genes; and 2) the coupling of G4 formation to transcription, such that all hG4-mediated activities operate in a transcription-dependent manner at the transcriptome scale. In general, the identification of hG4s greatly expands the prevalence and functionality of G4s in close association with transcription.

**Fig. 1.**
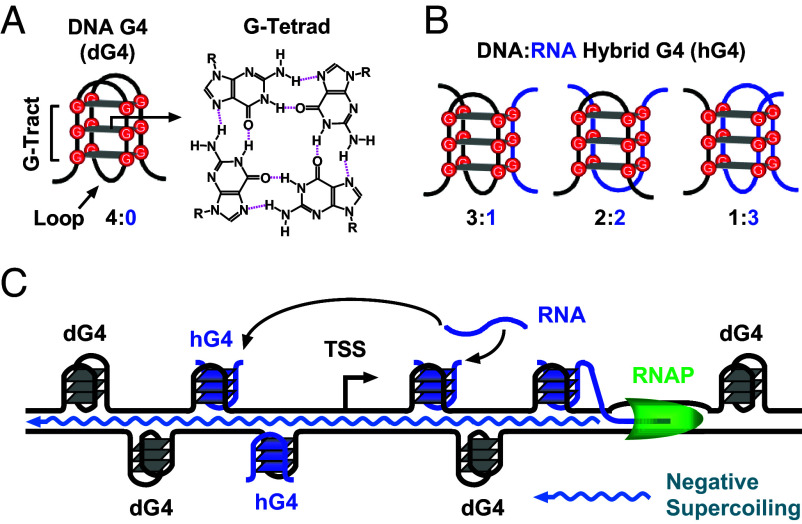
Intramolecular and intermolecular G4 structures and their formation in a DNA duplex transcribed in vitro. (*A*) Intramolecular G4 composed of four G-tracts with three G-tetrads. (*B*) DNA:RNA hybrid G4s can be formed in transcribed DNA by the joint participation of G-tracts from DNA and RNA with different G-tract stoichiometry. (*C*) G4 formation can be triggered by a transcribing RNAP on its upstream side through the propagation of negative supercoiling and on its downstream side by an approaching transcription bubble.

Guanine-rich nucleic acids have the ability to form four-stranded G-quadruplex (G4) structures in which four guanine tracts (G-tracts) are bundled by stacked guanine tetrads (G-tetrads) (Fig. 1*A*). Due to their diverse topology, physical stability, and specific genomic location, G4s play critical roles in various cellular processes, including replication, transcription, translation, and genome stability (1, 2). Putative G-quadruplex sequences (PQSs) are found throughout the genomes of both prokaryotic and eukaryotic organisms (3). Importantly, PQSs are not randomly distributed but are highly concentrated in regulatory regions, particularly in promoters of higher organisms, suggesting their involvement in the regulation of gene expression (4, 5). DNA G4 has been detected in human genome by a variety of techniques [see a recent review (6)]. Currently, our understanding of the structural forms of G4s in living cells is mostly extrapolated from in vitro studies. However, G4 formation in cells occurs in a completely different environment, which has been shown to cause G4s to behave differently in terms of kinetics, conformation, stability, and other properties than they do under simplistic in vitro conditions (7).

In principle, the four G-tracts of a G4 can originate from a single or multiple nucleic acid strands, resulting in either an intramolecular or intermolecular structure. Research in G4 biology has primarily focused on canonical intramolecular DNA or RNA G4s, where four G-tracts are supplied from a single strand (Fig. 1*A*), as highlighted in recent reviews (4, 5). Although G4s have been detected in the genomes of animal cells by various techniques (6, 8, 9), their structural form in living cells has not been determined. A decade ago, our in vitro studies demonstrated the formation of a specific type of G4s, termed DNA:RNA hybrid G4s (hG4s) (Fig. 1 *B* and *C*), in transcribed DNA. This was achieved by simultaneous recruitment of G-tracts from a nontemplate DNA strand and RNA transcripts (10, 11). In our studies (3, 10–18), we characterized hG4s in terms of their formation mechanism, kinetics, stability, structural diversity, competition with DNA G4s (dG4s), and other features. Interestingly, we found that hG4s can still form and even dominate in DNAs with four G-tracts, although these DNAs are theoretically capable of forming intramolecular dG4s by themselves (14). This phenomenon was later explained by in vitro studies showing that hG4s are more stable and fold faster than the corresponding dG4s (18–22). Although we have confirmed the formation of hG4s in living bacterial cells (14), it is still unclear whether such structures can form in eukaryotic cells.

The recruitment of G-tracts from RNA allows DNA to form a G4 with as few as one DNA G-tract, instead of four (Fig. 1*B*). This results in a significantly higher number of hG4 sites in a genome compared to dG4 sites (11). For example, in the yeast genome, the number of hG4 PQSs is over 3,300 times greater than that of dG4 PQSs, considering only those capable of forming a G4 with three or more G-tetrads. Previous in vitro studies have shown that hG4 formation is directly proportional to transcriptional activity (15, 23), indicating a tight coupling between hG4s and transcription. Therefore, hG4 formation represents a unique genotype that is significantly more abundant and likely plays a distinct role in conjunction with transcription. However, uncertainty about its existence has prevented researchers from exploring and understanding this unique genotype in the eukaryotic kingdoms. The answer to the question of whether hG4 can form in eukaryotic cells should reveal an in vivo reality, as well as a unique regulatory mechanism that has gone virtually unnoticed. In this study, we present evidence for and characterize the genome-wide formation of hG4s in the yeast genome and discuss its implications for genome-related cellular activities.

## Brief Background on G4 Formation In Vitro

Our group has performed most of the studies (3, 10–18) on hG4s (3, 10–22, 24, 25), including their discovery, identification, mechanism of formation, and effect on transcription in physiological environments. The formation of G4s is a dynamic process that can be driven by different mechanisms. On the nontemplate strand downstream of a moving RNA polymerase (RNAP), G4 formation is triggered when an approaching transcription bubble is 7 nucleotides (nts) away from a PQS (26) (Fig. 1*C*). Alternatively, a G4 can form behind a moving RNAP driven by a negative supercoiling wave (23, 27), which can propagate upward and induce G4 formation over a range of several thousand nts (27) (Fig. 1*C*). In the case of hG4s, they can begin to form during the second round of transcription when RNA produced on the template strand in the previous round is displaced to provide G-tracts (12). In addition, an hG4 can form on the upstream side of transcription with G-tracts in RNA produced by transcription on the downstream side (16). Compared to dG4s, hG4s have a faster folding rate and greater mechanical stability (18), allowing hG4s to dominate even when a DNA itself is capable of forming a dG4 (14). Taken together, these observations suggest that hG4s can be formed regardless of the genomic location of DNA G-tracts, as long as there is an adequate supply of RNA G-tracts from the cellular RNA pool.

## Results

### G4 Detection Based on Stop of Okazaki Fragment (OKF) Synthesis.

**Fig. 2.**
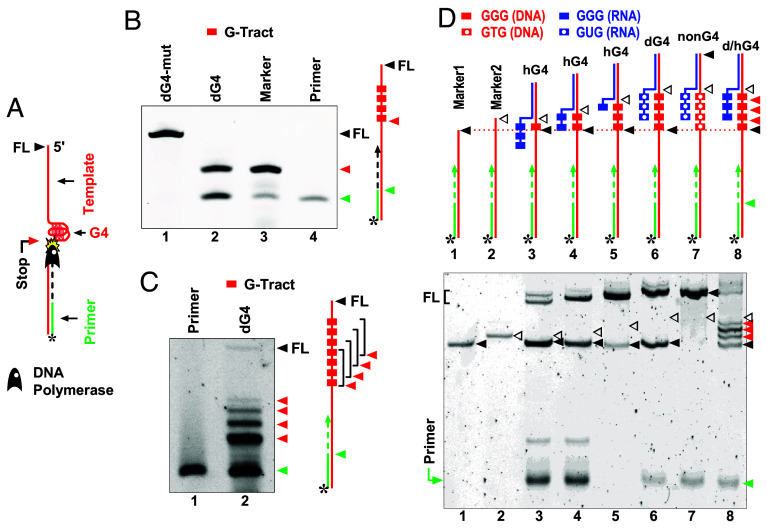
DNA polymerase stop assay with hG4 and dG4 in the template examined by denaturing gel electrophoresis. (*A*) Illustration of primer extension by DNA polymerase, where premature stop occurs at a G4 in addition to full-length (FL) extension. Extension products were visualized by the fluorescent dye FAM (*) at the 5′ end of the primer. (*B*) Primer extension with a mutated (lane 1) or a native PQS (lane 2) from between the yeast ATO3 and EFT2 genes. (*C*) Primer extension stopped at four alternative positions (red arrowheads) with a PQS of seven G_3_ tracts (lane 2) capable of forming a dG4 at the four alternative positions. (*D*) Primer extension stopped at hG4s in the template. The template (red line) was annealed with an RNA oligomer (blue line), with or without G-tracts.

Protein translocation along a DNA strand is impeded by a G4 structure (28), which has led to the development and widespread use of a polymerase stop assay (29, 30) for the in vitro detection of G4s in both DNA (29–31) and RNA (32, 33). In this method, a short DNA primer is annealed to a target strand and extended by DNA polymerase. The extension stops upon encountering a G4 to signal the presence of the G4 (Fig. 2*A*). As shown in Fig. 2*B* (lane 2, red arrowhead), a yeast DNA G4 halted the progress of the DNA polymerase. In contrast, the reaction reached its full length when the PQS was mutated to prevent G4 formation (lane 1). In Fig. 2*C*, the DNA template contained seven consecutive G-tracts, allowing for the formation of only one dG4 at four alternative positions (34). Consequently, four DNA pausing bands corresponding to the dG4 at the four alternative positions are observed.

We tested whether the method could also detect hG4s. We used DNA templates with different numbers of G_3_ tracts to form dG4 or hG4 at different DNA:RNA G-tract ratios (Fig. 2 *D*, *Top*), with an RNA oligomer annealed to the DNA template as described (12). As shown in Fig. 2 *D*, *Bottom*, primer extensions paused before both hG4s and dG4 (lanes 3 to 6, black arrowheads). The pause disappeared when mutations were introduced into the G-tracts to prevent G4 formation (lane 7). With a supply of three RNA G-tracts in lane 8, theoretically one hG4 could form at each of the four DNA G-tracts. As predicted, four pause bands are observed. The multiple pauses here indicate that the DNA intended to form hG4 when RNA G-tracts were present. Otherwise, one would expect a single pausing band at the first DNA G-tract encountered by the polymerase. Collectively, these results demonstrate that the DNA polymerase stop assay can also be used to detect hG4 formation. In this assay, primer extension did not stop at the DNA:RNA hybrid duplex (lanes 3 to 8, open arrowheads), the core structure of R-loops.

To detect G4 formation in living yeast cells, we used the in vitro polymerase stop principle to monitor the progress of Okazaki fragment (OKF) synthesis primed by RNA instead of DNA. OKFs are short DNA fragments generated within a replication fork on the lagging strand during DNA replication (35). In recent years, OKF synthesis in yeast has been extensively studied using the OK-Seq technique on other topics in several independent studies (36–40), which provided us with the original OK-Seq data for this work.

### Retardation of OKF Synthesis by PQSs.

**Fig. 3.**
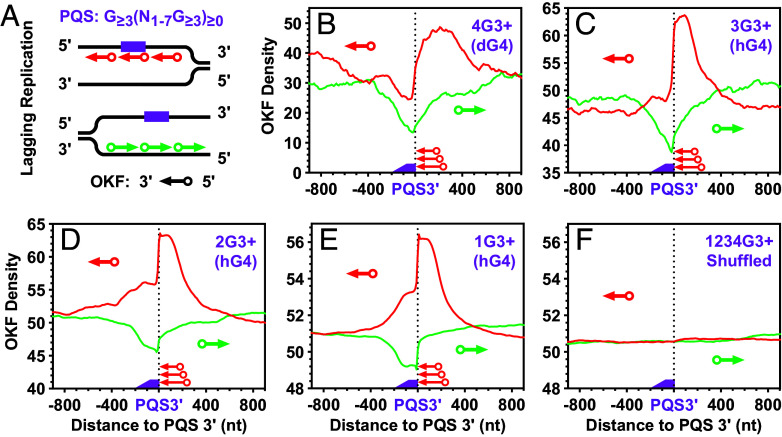
Distribution of OKFs at the 3′ end of PQSs with 1 to 4 or more G_≥3_ tracts that could form G4s of three or more G-tetrads. (*A*) Scheme of profiling on the PQS-bearing strand and the strand opposite to a PQS. (*B*–*E*) Distribution at PQSs capable of forming (*B*) dG4s or (*C*–*E*) hG4s. (*F*) Distribution at randomly shuffled PQSs. Original data for this and OKF-related figures are from GSE115897 unless otherwise noted. The “3+” sign indicates that the G-tracts in the PQS had three or more consecutive guanines; the digit before the “G” indicates the number of G-tracts in the PQS; text in parentheses indicates the type of G4 the PQS can form.

A stable G4 usually has three or more G-tetrads. We first identified PQSs in the yeast genome and classified them into four groups, 4G3+, 3G3+, 2G3+, and 1G3+, based on the number of G_≥3_ tracts. While the 4G3+ PQSs can form dG4s by themselves, the 3G3+, 2G3+, and 1G3+ PQSs have the potential to form hG4s by recruiting additional G-tracts from RNA. We then profiled the distribution of OKFs relative to the 3′ ends of each PQS group (Fig. 3*A*). Regardless of the G4 type that the PQSs could form, all profiles showed a positive peak downstream of the PQSs on the PQS-bearing strands (Fig. 3 *B*–*E*, red lines). These positive peaks showed an abrupt decrease at the 3′ end of the PQSs, suggesting that OKF synthesis was intercepted at the 3′ front of the G4s in the 4G3+ PQSs (Fig. 3*B*) and hG4s in the 3G3+, 2G3+, and 1G3+ PQSs (Fig. 3 *C*–*E*), respectively, as in the in vitro polymerase stop assay (Fig. 2) (29).

In contrast to the PQSs, the corresponding complementary cytosine-rich (C-rich) motifs were instead associated with a negative OKF peak (Fig. 3 *B*–*E*, green lines), because they do not form G4s, while their flanking regions might have scattered background G4 formation. On the other hand, G4 formation prevents duplex annealing, which might facilitate OKF synthesis through this C-rich region. The two oppositely polarized OKF peaks were specifically associated with PQSs, as they both disappeared when the coordinates of the PQSs were randomly shuffled across the genome and the OKF signal was reprofiled over the resulting fake PQSs (Fig. 3*F*). Similar profiles were obtained with four additional independent datasets from the GEO database (*SI Appendix*, Figs. S1–S4), suggesting that the retardation of OKF synthesis at the 3′ end of PQSs is a universal phenomenon.

Two other structures, R-loops and i-motifs, may hamper OKF synthesis. An R-loop is formed when a nascent RNA transcript hybridizes to the template DNA strand (41). According to a study in human cells, the R-loop is preferentially formed with a G-rich RNA transcript annealing to the C-rich strand (42). In this case, one would expect a preferential retardation of OKF synthesis on the C-rich than the PQS-bearing strands, which contradicts our observation. In addition, an R-loop spans a region much larger than the size of a PQS (12), so it does not explain the sharp alignment of OKFs at the 3′ ends of PQSs. Furthermore, DNA synthesis in Fig. 2 did not show retardation at the DNA:RNA heteroduplex. As for the i-motif, the formation of this structure requires an acidic pH of 4.5 to 6.5 (43), which is unfavorable in eukaryotic cells, since the reported pH values are 7.2 for the cytosol and 7.2 for the nucleus (44). If i-motifs could form, one would also expect a preferential retardation of OKF synthesis on the C-rich other than on the PQS-bearing strands. Therefore, the unique retardation pattern of OKF synthesis can hardly be explained by these two structures.

To further verify the G-tract dependence, we performed a similar analysis using motifs in which the guanines were substituted with adenines. In this case, a negative peak was observed on both DNA strands for the OKF (*SI Appendix*, Fig. S5). This result was expected since the A/T-rich motifs lacked the ability to form G4, while their flanking regions had a scattered background of G4 formation. Introducing a mutation in the middle of a G-tract effectively disrupts G4 formation (Fig. 2) (11, 45). We also examined motifs containing one or more GNG tracts, where N represents any nucleotide other than G. Since these motifs were either unable to form G4, they also exhibited a negative OKF peak on both DNA strands (*SI Appendix*, Fig. S6). As expected, shuffling these two types of motifs resulted in the disappearance of the negative peaks (*SI Appendix*, Figs. S5*F* and S6*F*). Taken together, these results (Fig. 3 and *SI Appendix*, Figs. S1–S6) support the formation of hG4s in the PQSs.

### Detection of G4 Formation with G4 Probe (G4P) Protein.

**Fig. 4.**
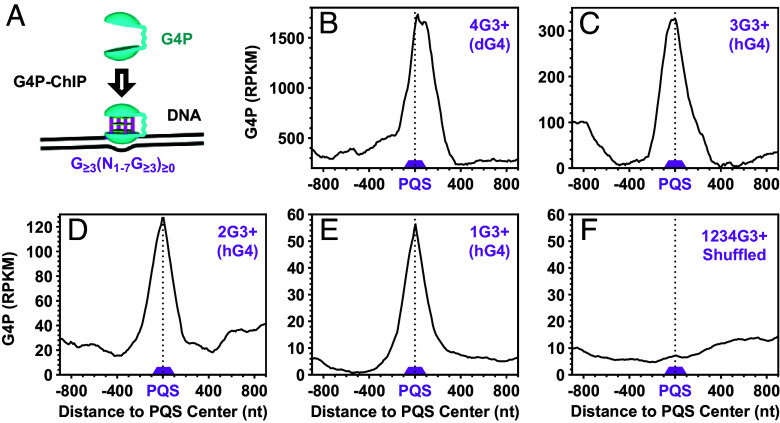
Binding profiles of G4P at PQSs with 1 to 4 or more G_≥3_ tracts that could form G4s of three or more G-tetrads. (*A*) Scheme of G4 detection by G4P ChIP-Seq. (*B*–*E*) Enrichment at PQSs capable of forming (*B*) dG4s or (*C*–*E*) hG4s. (*F*) Distribution at randomly shuffled PQSs.

The G-tract-dependent arrest of OKF synthesis at the PQSs provides evidence for the formation of G4 structures in these regions (29). We next expressed a small G4P protein in yeast cells and performed G4P Chromatin immunoprecipitation followed by sequencing (ChIP-Seq) to examine G4 formation (Fig. 4), following an approach used in human and other animal cells (8). The G4P, with its two G4-binding domains and a molecular weight of only 6.7 kDa (Fig. 4*A*), exhibits high specificity for different G4s (8), including hG4s (*SI Appendix*, Fig. S7). Consistent with the OKF signal (Fig. 3), the G4P showed enrichment at the PQSs, which could form either dG4s (Fig. 4*B*) or hG4s (Fig. 4 *C*–*E*). This enrichment disappeared when the coordinates of the PQSs were randomly shuffled, and the G4P signal was reprofiled (Fig. 4*F*). We also analyzed the G4P signal over the A/T-rich and GNG motifs. Consistent with the OKF signals at these motifs (*SI Appendix*, Figs. S5 and S6), G4P also showed a negative peak (*SI Appendix*, Figs. S8 and S9), further supporting the formation of hG4 and dG4 in the PQSs.

### Detection of G4 Formation with G4access.

**Fig. 5.**
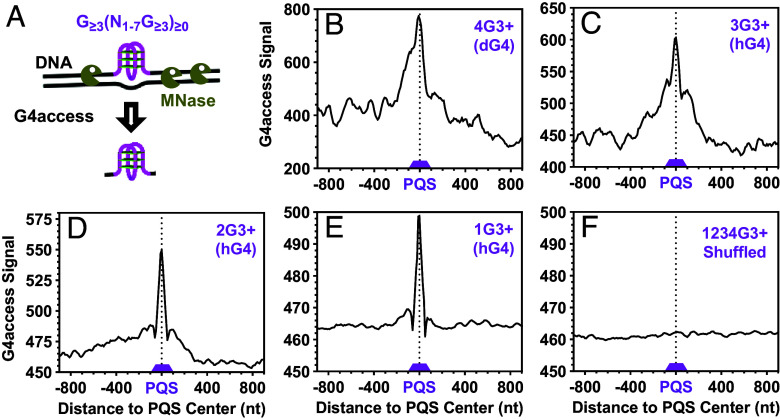
Distribution of G4access signals at PQSs with 1 to 4 or more G_≥3_ tracts that could form G4s of three or more G-tetrads. (*A*) Scheme of G4 detection by G4access. (*B*–*E*) Enrichment at PQSs capable of forming (*B*) dG4s or (*C*–*E*) hG4s. (*F*) Distribution at randomly shuffled PQSs.

The profiling of OKF synthesis (Fig. 3) and the probing with G4P (Fig. 4) both supported the formation of hG4 and dG4 at the PQSs. To further validate hG4 formation, we turned to a newly developed technique, G4access, which identifies G4s that are resistant to micrococcal nuclease (MNase) digestion in open chromatin (9). Profiles of the G4access signals over the PQSs also showed enrichment at the PQSs, which could form either dG4s (Fig. 5*B*) or hG4s (Fig. 5 *C*–*E*). The enrichment also disappeared when the coordinates of the PQSs were shuffled to random locations (Fig. 5*F*). When the profiles were calculated over the A/T-rich (*SI Appendix*, Fig. S10) and GNG (*SI Appendix*, Fig. S11) motifs, little or no enrichment was observed. Taken together, the formation of hG4 and dG4 was also supported by the G4access technique.

### Formation of hG4s and dG4s of Two G-Tetrad Layers.

While the G4 community has primarily focused on stable G4 structures with three or more G-tetrads, our previous study revealed the formation of hG4s with only two G-tetrads in DNA duplexes transcribed in vitro (10). These hG4s are less stable than those with three or more G-tetrads (10, 15). We were intrigued to investigate whether such hG4s could also form in living cells. Therefore, we analyzed OKF, G4P, and G4access signals around PQSs containing only GG tracts. The results showed an accumulation of OKF (*SI Appendix*, Fig. S12), and an enrichment of G4P (*SI Appendix*, Fig. S13) and G4access signals (*SI Appendix*, Fig. S14) at the PQSs, providing evidence for the formation of hG4s and dG4s of two G-tetrad layers in the genome. Based on this finding, our subsequent analysis of G4 will include those with two G-tetrads.

We have now used three completely independent techniques to detect the formation of hG4 and dG4. Our previous studies have shown that the formation of hG4 in DNA duplexes transcribed in vitro is positively proportional to the number of DNA G-tracts (11, 14), which is expected because more G-tracts provide a greater chance for G4 formation. We examined the G4 formation detected by the three techniques for its dependence on the number of DNA G-tracts (*SI Appendix*, Fig. S15). As we can see, the results are consistent with the in vitro observation. The much greater peak of G4P for 4G3+ than for the other PQSs (*SI Appendix*, Fig. S15, *Top*-*Middle* panel) probably reflects random variation due to the much smaller 4G3+ counts in the genome.

### Presence of RNA G-Tracts in hG4s.

**Fig. 6.**
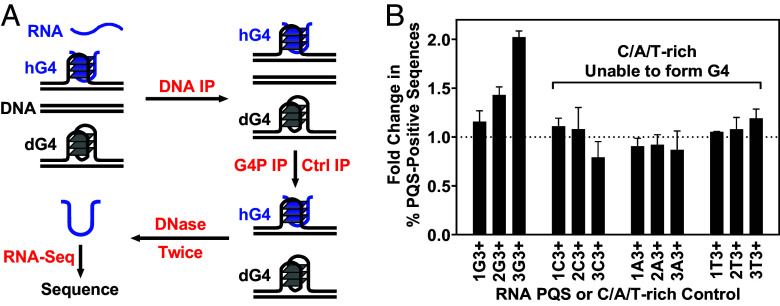
Detection of RNA G-tracts in hG4s with three or more G-tetrads. (*A*) Workflow of two-step IP and RNA sequencing (RNA-Seq). Precipitation of G4 with an anti-FLAG antibody (G4P IP) was performed in parallel with a nonspecific antibody as control (Ctrl IP). (*B*) Change in percentage of G-tract-positive RNA sequences in G4P-bound DNA compared to control, expressed as mean ± SEM of three biological replicates.

In the above results, the stalling of OKF synthesis next to the PQSs (Fig. 3 and *SI Appendix*, Figs. S1–S4 and S12), the G4P (Fig. 4 and *SI Appendix*, Fig. S13) and G4access signals (Fig. 5 and *SI Appendix*, Fig. S14) enrichment at the PQSs provided clear support for the formation of hG4s at the PQSs with less than four G-tracts. Since these motifs alone were unable to form G4s, the involvement of RNA G-tracts is expected. To check this out, we performed a two-step immunoprecipitation (IP) to sequence the RNA in the G4s (Fig. 6*A*). A DNA antibody was used to capture cross-linked DNA fragments that could contain dG4s, hG4s, or R-loops. The captured DNA was then further precipitated using a G4P-binding antibody or a nonspecific antibody of the same origin as a control. The DNA in the precipitates was digested with DNase, and the remaining RNA was sequenced. As shown in Fig. 6*B*, RNAs were detected and those with 1 to 3 G-tracts were enriched compared to the non-G4-forming C/A/T-rich controls. In addition, the degree of enrichment was positively correlated with the number of RNA G-tracts, which is consistent with the known fact that more G-tracts have a greater tendency to participate in hG4 formation (11). Similar result was obtained when RNA GG tracts and the corresponding control motifs were included (*SI Appendix*, Fig. S16). In this case, a lower enrichment was observed because G4s with shorter G-tracts are relatively much less stable (10, 15).

### Interception of OKF Synthesis by hG4s at PQSs.

**Fig. 7.**
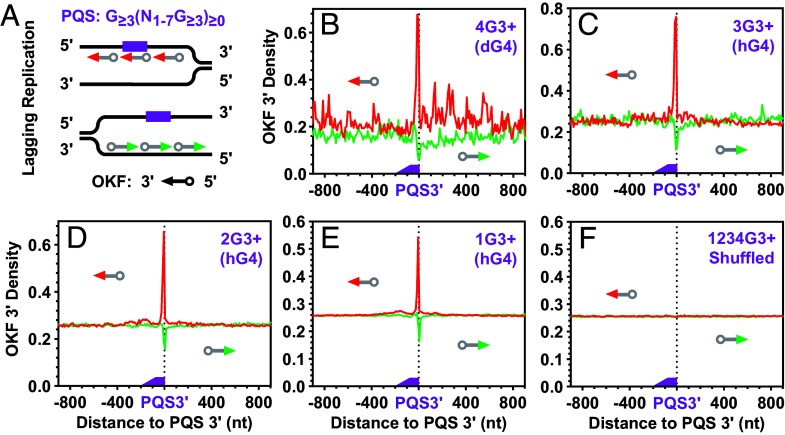
Distribution of OKF 3′ ends at the 3′ end of PQSs with 1 to 4 or more G_≥3_ tracts that could form G4s of three or more G-tetrads. (*A*) Scheme of profiling on PQS-bearing strand and strand opposite to PQS. (*B*–*E*) Distribution at PQSs capable of forming (*B*) dG4s or (*C*–*E*) hG4s. (*F*) Distribution at randomly shuffled PQSs. Note that the body and tail of the arrows are grayed out to indicate that only the 3′ end coordinates of the OKFs were profiled.

**Fig. 8.**
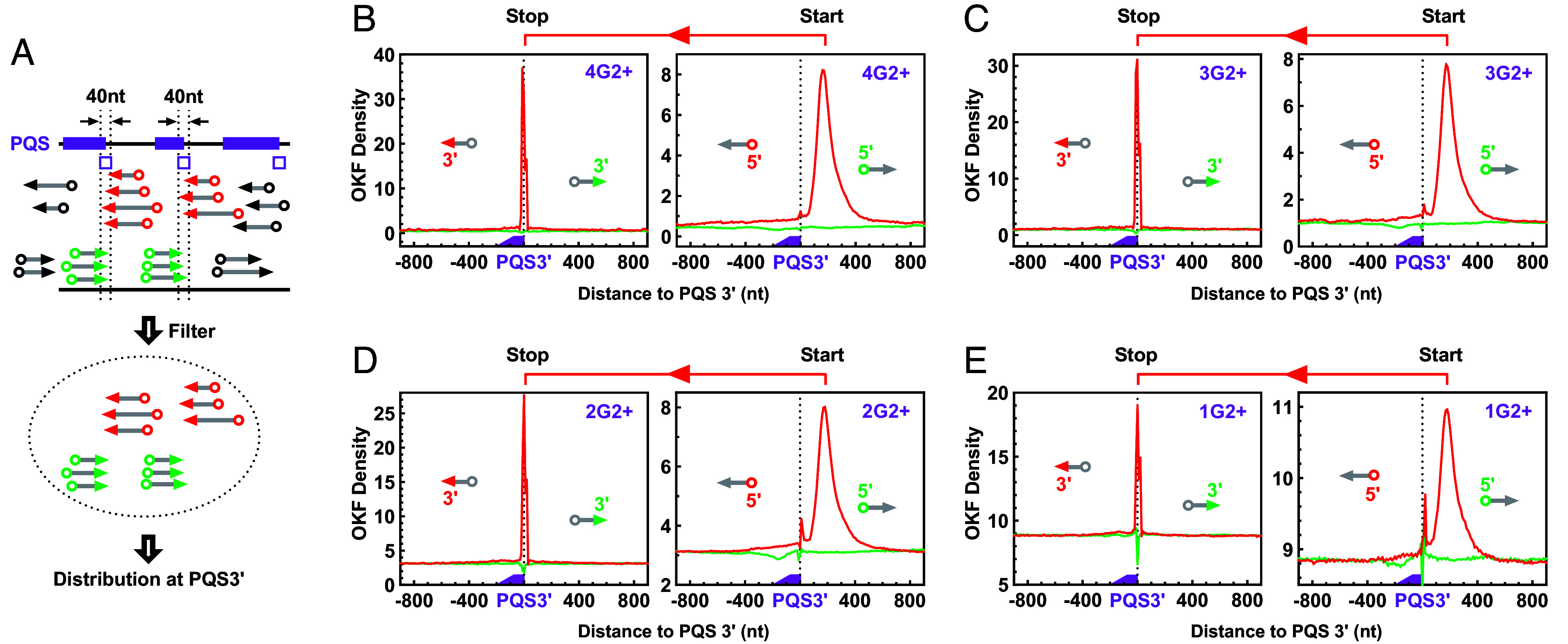
Interception of OKF syntheses at the 3′ end of PQSs. (*A*) Scheme of filtering for OKFs whose 3′ end was within the ±20 nts region (open box) of the PQS 3′ end. (*B*–*E*) Interception of OKF syntheses at the 3′ end of PQSs capable of forming (*B*) dG4s or (*C*–*E*) hG4s of two or more G-tetrads.

The OKFs are of different lengths, which was a source of variation that contributed to the OKF profiles (Fig. 3 and *SI Appendix*, Figs. S1–S4 and S12). To determine the exact termination position of the OKFs, we extracted the coordinates of the 3′ end of the OKFs and plotted their distribution across the PQSs (Fig. 7*A* and *SI Appendix*, Fig. S17*A*, arrowhead). A sharp peak was observed at the 3′ end of the PQSs, regardless of whether the PQSs were able to form dG4 or hG4 with two (*SI Appendix*, Fig. S17 *B*–*E*) or more (Fig. 7 *B*–*E*) G-tetrads. This sharp peak suggests that the syntheses of OKFs were intercepted at the 3′ side of the G4s in the PQSs. If no G4s were formed, then the 3′ end of these OKFs would terminate randomly instead. To validate the interception, we collected OKFs whose 3′ end was within a range of ±20 nts from the 3′ end of the PQSs and analyzed the location of both their 5′ and 3′ ends (Fig. 8*A*). The results showed that the corresponding 5′ ends of these OKFs were distributed over a wide range of approximately 500 nts downstream of the PQSs (Fig. 8 *B*–*E*). This feature indicates that the syntheses of these OKFs were initiated from a broad region and, according to their sharp 3′ end peak at the 3′ end of the PQSs, were abruptly interrupted by the G4s in the PQSs. On the other hand, the sharp peak of the OKF 3′ end with improved background (Fig. 7 and *SI Appendix*, Fig. S17) makes it a much clearer indicator of G4 formation.

### The Structural Form of hG4s.

In the polymerase stop assay, the number of stop sites is determined by the number of alternative G4s that can form with the available G-tracts (14) (Fig. 2*C*). In the case of a single DNA G-tract, only one hG4 can form with three additional RNA G-tracts, so a single stop is expected. For a DNA PQS with four G-tracts, only one stop would occur if a single dG4 forms by consuming all the four G-tracts (Fig. 2*B*). In contrast, when hG4s form, a maximum of four stops is expected, with each hG4 may having one G-tract from DNA and three from RNA. This principle has allowed us to distinguish hG4 from dG4 in transcribed DNA both in vitro and in bacteria (14). Here, we used the same approach to further identify the composition of G4s based on the number of stop signals of OKF synthesis.

**Fig. 9.**
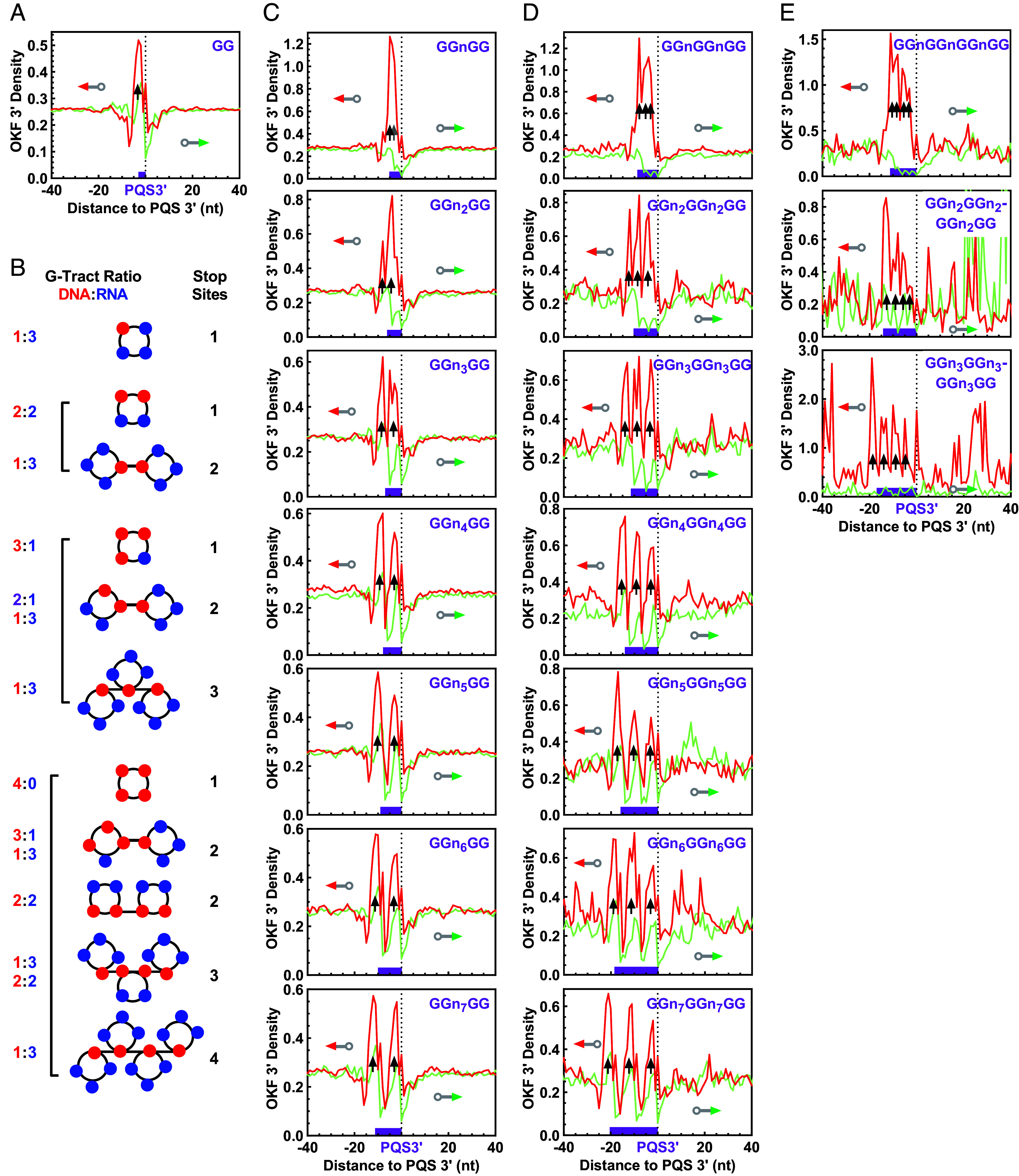
Distribution of OKF 3′ ends at the 3′ end of PQSs with different number of GG tracts showing the involvement of DNA G-tracts in hG4 formation. (*A*) One GG tract. (*B*) Examples of combinations of DNA and RNA G-tracts in hG4 formation. (*C*) Two GG tracts. (*D*) Three GG tracts. (*E*) Four GG tracts. “n” denotes any nucleotide, but not G if adjacent to G. Bin size: 1 nt.

In Fig. 9, we examined the extension of OKFs at single nucleotide resolution, spanning an 80 nt region centered on PQSs containing 1 to 4 GG tracts, each with loops of identical size. For a single GG tract, a single prominent OKF 3′ end peak was observed, indicating the formation of a single hG4 consisting of one GG tract from DNA and three G-tracts from RNA (Fig. 9*A*). In Fig. 9*B*, examples of different combinations of G-tracts from DNA and RNA for hG4 formation and the corresponding expected stops in OKF synthesis are listed. In Fig. 9*C*, OKF syntheses around two GG tracts resulted in two major peaks that became more prominent when the loop size was increased from 1 to 7 nts for better resolution. This observation was consistent with the formation of two hG4s, each composed of one GG tract from DNA and three G-tracts from RNA. The same rule extended to the PQSs with three GG tracts, for which three peaks were detected accordingly (Fig. 9*D*).

**Fig. 10.**
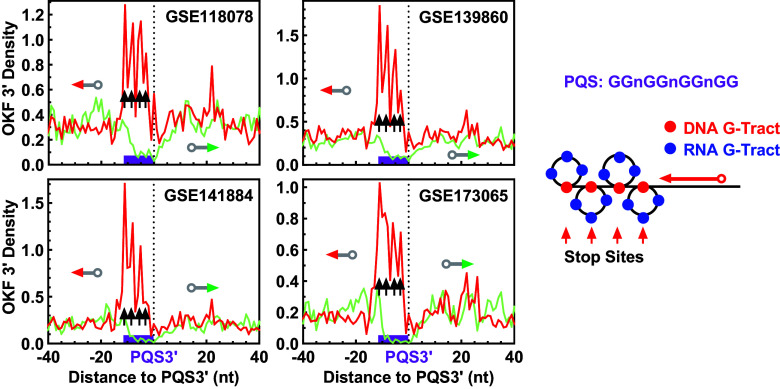
Distribution of OKF 3′ ends at the 3′ end of PQSs with four GG tracts and 1-nt loops showing the involvement of DNA G-tracts in hG4 formation. The source of the original OK-Seq data is indicated in the panels. “n” denotes any nucleotide other than G. Bin size: 1 nt.

A PQS with four GG tracts would yield a single OKF 3′ peak if all GG tracts were used to form a single dG4. Interestingly, these PQSs instead showed four peaks, indicating the formation of four hG4s (Fig. 9*E*) as in the in vitro DNA polymerase stop assay (Fig. 2*D*, lane 8). This behavior was further confirmed in four additional datasets from independent studies (Fig. 10), suggesting that a DNR:RNA G-tract combination of 1:3 was used to form each of the four hG4s (Fig. 10, scheme at *Right*), although other forms of combinations could not be excluded. Similar results were obtained for the GGG (*SI Appendix*, Fig. S18) and GGGG (*SI Appendix*, Fig. S19) DNA tracts, all supporting the capability of forming an hG4s with a single G-tract. Apparently, these discrete peaks can hardly be explained by an R-loop.

The positive peaks of the OKF 3′ ends associated with PQSs of different numbers of GG, GGG, and GGGG tracts (Figs. 9 and 10 and *SI Appendix*, Figs. S18 and S19) all disappeared when the locations of the PQSs were randomly shuffled (*SI Appendix*, Figs. S20–S23), implying that the observation was PQS-specific. The positive peaks also disappeared when the 3′ ends of OKF were profiled over motifs with different numbers of AA, AAA, and AAAA tracts (*SI Appendix*, Figs. S24–S27). In these cases, negative peaks corresponding to the number of A-tracts were often seen (e.g., *SI Appendix*, Fig. S24), possibly due to the inability of the A-tracts to form G4s. Collectively, the discrete pausing of OKF syntheses at the PQSs is well explained by hG4 formation.

Taken together, our results in this section provide two important findings. First, even a single GG tract in the yeast genome can form an hG4. Second, a PQS with four G-tracts can still form hG4s, although it can simply form an intramolecular dG4 by itself. Judging from the well-resolved four dominating peaks (Figs. 9*E* and 10), it can be inferred that the hG4s were the major form of G4s. If not, a single prominent OKF 3′ peak would be expected at the first G-tract from the downstream side of the PQSs. A PQS in a chromosome is constrained in a more rigid DNA duplex, making the folding of G-tracts more challenging compared to a more flexible RNA strand. The dominance of hG4 formation in such cases was observed in our previous studies using linear duplex and plasmid DNA transcribed in vitro with T7 RNAP (14, 18), which was explained by the observation that hG4 formation is favored by faster kinetics and greater stability (18).

### Formation of hG4 in Orphan PQSs.

**Fig. 11.**
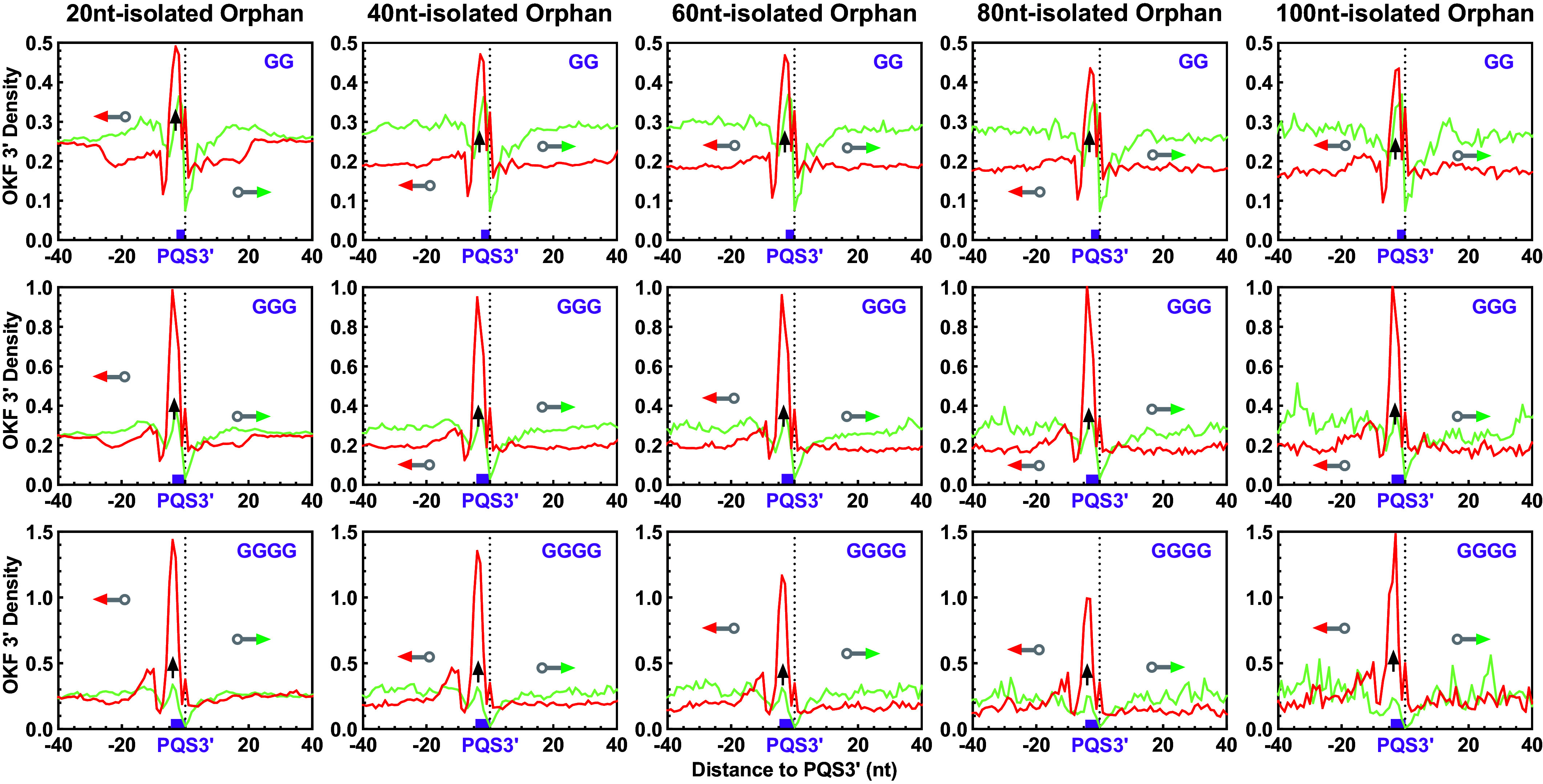
hG4 formation at orphan PQSs isolated from neighboring G-tracts by different numbers of nucleotides, detected by OKF 3′ end pausing at single GG, GGG, GGGG tracts. Bin size: 1 nt.

A PQS is defined by a consensus that limits the loop between G-tracts to a maximum of seven nts, a rule that is generally accepted by the G4 community. However, when considering a PQS with fewer than four G-tracts, it is possible that neighboring G-tracts beyond the loop limit may be recruited to form a dG4 rather than an hG4. To address this concern, we identified three groups of PQSs carrying a single run of GG, GGG, and GGGG, respectively, separated from any other G_≥2_ tract by at least 20 to 100 nts. These G-tracts, termed “orphan G-tracts,” were analyzed for OKF stop signal (Fig. 11).

For all these G-tracts, a single prominent OKF 3′ end peak was detected as an indication of hG4 formation. In particular, the peak height remained constant as the isolation flanks were extended from 20 to 100 nts (Fig. 11, panels from *Left* to *Right*), arguing against the possibility of dG4 formation. Otherwise, a decrease in G4 formation with increasing isolation size would be expected (46). In contrast, the peak height increased as the G-tract size increased from 2 to 4 nts (Fig. 11, panels from *Top* to *Bottom*), consistent with the well-documented fact that G4s with longer G-tracts are more stable (47).

The formation of hG4s was further confirmed by the enrichment of G4P (*SI Appendix*, Fig. S28*A*), as indicated by a G4P peak at most of the orphan G-tracts. The number of such orphan G-tracts rapidly decreased with increasing isolation size, leading to a degradation of the G4P signal. To improve the signal-to-noise ratio, we analyzed orphan PQSs with 1-3 G_≥2_ tracts that could not form a dG4 by themselves. In this case, a clearer G4P peak was detected for all five isolation sizes (*SI Appendix*, Fig. S28*B*). Furthermore, the formation of hG4s was also verified by the G4access approach, in which the peak amplitude mostly increased with increasing G-tract size as expected (*SI Appendix*, Fig. S29, panels from *Top* to *Bottom*). Unlike the OKF 3′ peaks, the amplitude of the G4access peaks increased with increasing isolation size (panels from *Left* to *Right*). Since G4s are resistant to MNase digestion, a larger G4-free isolation flank is expected to facilitate digestion, releasing more G4s to be detected. Taken together, these results from the orphan G-tracts confirmed the ability of a PQS with less than four G-tracts to form an hG4.

### Survey of hG4 and dG4 Sites in the Yeast Genome.

**Fig. 12.**
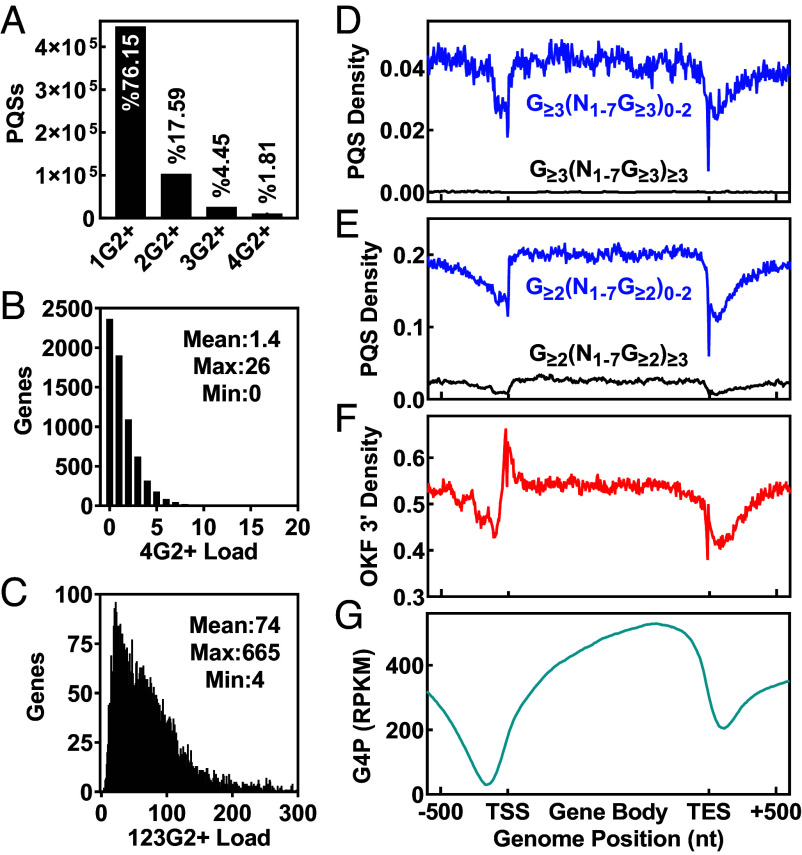
Overview of potential hG4 sites in the yeast genome and genes. (*A*) Counts of total PQS with different numbers of G-tracts. (*B*) PQS capable of forming dG4s in genes. (*C*) PQS capable of forming only hG4s in genes. (*D*) Distribution of PQS capable of forming only hG4s (blue line) and dG4s (black line) of three or more G-tetrads in genes. (*E*) Distribution of PQS capable of forming only hG4s (blue line) and dG4s (black line) of only two G-tetrads across genes. (*F*) Distribution of 3′ ends of OKFs across genes. (*G*) Binding of G4P across genes.

Our analyses have revealed a distinctive picture that goes far beyond our previous perception of the nature and extent of G4 formation in the yeast genome. To get an overview, we surveyed the abundance of potential G4 sites in the genome and genes capable of forming either dG4s or hG4s with two or more G-tetrads. Since promoter activities are mostly concentrated in the 120 nts region upstream of TSSs in yeast (48, 49), we merged this region with the gene body for the analysis. According to the canonical consensus, the yeast genome can only form up to 38 dG4s with three G-tetrads. The identification of hG4 formation increased the total number of G4 sites to 587,694, a >15,000-fold increase, of which canonical dG4 sites with two or more G-tetrads account for less than 2% (Fig. 12*A*). Furthermore, hG4 sites are more abundant in genes, with a >50-fold higher abundance compared to dG4 sites (Fig. 12*B* versus Fig. 12*C*), highlighting the dominance and role of hG4s in transcriptional regulation.

We also profiled the distribution of PQS, OKF, and G4P signals across genes. The results (Fig. 12 *D* and *E*) showed that PQSs had a similar density throughout the genome, with a notable decrease near TSSs and transcription end sites (TESs). In contrast, the OKF 3′ signal showed its highest peak at TSSs (Fig. 12*F*), probably due to higher production of RNA transcripts in this region. However, G4P binding instead showed a gradual decrease toward the TSSs (Fig. 12*G*), which might be attributed to the increased activity of protein interaction and translocation associated with transcription initiation. The discrepancy between OKF 3′ and G4P in their distribution pattern suggests that OKF 3′ may be more sensitive and efficient than G4P in detecting genomic G4 formation.

## Discussion

In conclusion, we have successfully used three techniques, the in vivo version of the polymerase stop assay with OK-Seq, the G4-binding protein G4P (8), and the G4access approach (9), each based on a different principle, to orthogonally detect the formation of hG4s in the yeast genome (*SI Appendix*, Fig. S30), providing evidence for their existence and dominance in living eukaryotic cells. Together with our previous study demonstrating the formation of hG4s in bacterial cells, our work suggests that the dominant and genome-wide formation of DNA:RNA hG4s is a universal phenomenon in both the prokaryotic and eukaryotic kingdoms. The biological functions of G4s are diverse (50, 51), and hG4s have several distinctive characteristics compared to canonical dG4s. First, hG4 sites are significantly more abundant because hG4s can form with as few as one DNA G-tract. Second, hG4s can also form and dominate in regions where DNA itself can form dG4s. Third, the different combinations of G-tracts of DNA and RNA G-tracts diversify the structural variability of hG4s (Fig. 9*B*) in many ways (14). Most importantly, the tight coupling between hG4 formation and transcription (15, 23) allows hG4s to mediate genome-wide transcription-dependent regulation.

dG4s are difficult to form in naturally supercoiled DNA duplexes (52–54). G4 formation in living cells is associated with negative supercoiling (8), demonstrating that melting of the DNA helix to single-stranded form is required. Such melting readily occurs in processes such as transcription and replication, allowing G4s to play a role in such cellular activities. The folding conformation of a G4 is controlled by either kinetics or thermodynamics, resulting in different conformations (55). This may also apply to the equilibrium or competition between the formation of dG4 and hG4 in a PQS, which is expected to add further structural diversity to a genome. How this is regulated and how it responds to nearby ongoing DNA processes deserves further investigation.

G4s have been implicated in transcriptional regulation (56), with hG4s proposed to serve as constitutional cis-regulatory elements (11). Transcription-dependent hG4 formation may play a unique role in establishing a feedback loop for transcriptional regulation. G4s act as “roadblocks” that impede the translocation of proteins along a DNA strand (28, 57), including RNAP (11, 14, 58) and DNA polymerase, as demonstrated in this work. As a result, the presence of a G4 within a gene body results in transcriptional repression under both in vitro and in vivo conditions (11, 14, 59). An increase in transcription will lead to higher RNA levels and consequently higher hG4 formation, which in turn will lead to greater transcriptional repression. Given the widespread occurrence of hG4 formation in genes, this negative feedback may well serve as a common mechanism for maintaining transcriptional homeostasis and stability.

On the other hand, our study provides insight into the direct effect of hG4s on DNA replication. The stalling of DNA polymerase progression that we observed demonstrates how hG4s, acting as “roadblocks” on such a large genomic scale, can generally affect protein translocation along a DNA strand, a crucial event in genomic metabolism. Such effects may contribute to genome instability and explain why G4 stabilizing ligands cause DNA damage in a replication-dependent manner (60). In reality, an hG4 has the potential to affect any protein-DNA recognition or interaction, such as binding, diffusion, and translocation. PQSs with less than four G-tracts cause premature transcription termination under in vitro conditions, accompanied by a reduction in full-length transcript (11, 14). Plasmids containing such PQS also showed reduced expression when transfected into human cells (11). Transcriptomic analysis of human tissues showed that genes with high expression levels tended to have low numbers of h/dG4 PQSs, and those genes with high numbers of h/dG4 PQSs tended to have low expression levels (11). These observations support the formation of hG4s and demonstrate their involvement in replication and transcription.

In addition, the formation of hG4s of only two G-tetrads greatly diversifies the stability of G4 structures. Previous in vitro studies have shown that hG4s with two G-tetrads have a lifetime of minutes (10), while those with three G-tetrads can last for hours (15) if left undisturbed. Thus, these two sets of structures may play different roles, with the former being more responsive and the latter more persistent. Since the involvement of RNA G-tracts promotes G4 formation in both kinetics and stability (18), the combination of different ratios of DNA:RNA G-tracts in an hG4 should add another source of diversity.

Furthermore, the formation of hG4s may have played a role in evolution. PQSs capable of forming a dG4 are progressively selected during evolution, resulting in a higher frequency of these structures in higher species (3). From this perspective, the formation of hG4s in PQSs with 1 to 3 G-tracts of as few as two guanines could provide a smooth and necessary transition to facilitate the adaptation and selection of intramolecular dG4s. Without this gradual transition, the abrupt transition from nothing to the formation of canonical dG4s of four G-tracts would be difficult to justify.

Technically, the detection of G4s in living cells is challenging because G4s are dynamic structures that fold and unfold over time. Our work establishes OKF synthesis analysis as the only method that not only detects G4s but also distinguishes their structural form in unperturbed living cell genomes. G4 formation requires the opening of a DNA duplex, and our previous work has shown that G4 formation can be triggered by an approaching transcriptional bubble (26). A similar mechanism may also operate in a moving replication fork, where OKF synthesis and G4 formation occur simultaneously in close proximity, resulting in increased sensitivity for G4 detection. However, this mechanism may preferentially detect G4s in replicating DNA. It is important to note that transcription is an important driver of G4 formation (8). It is possible that G4s formed in regions that are actively transcribed but not replicated may be missed. Therefore, hG4s may form more extensively and abundantly in a genome than reflected by OKF synthesis.

## Materials and Methods

For polymerase stop assay, DNA:RNA constructs (*SI Appendix*, Table S1) were assembled (12) and primer extension was carried out using Bsu DNA polymerase, large fragment (NEB, USA) as previously described (29, 30). Detailed description of materials and methods, including electrophoretic mobility shift assay, data from public repositories, identification of PQS and control motifs in genome, identification of Orphan PQSs, plasmid construction for G4P ChIP-Seq, distribution of OKFs, G4P, and G4access signals across PQSs, and survey of PQSs in genes can be found in *SI Appendix*, *Materials and Methods*.

## Supplementary Material

Appendix 01 (PDF)

## Data Availability

Previously published data were used for this work (GSE115897 (36), GSE173065 (37), GSE141884 (38), GSE139860 (39), GSE118078 (40), GSE187007 (9) from GEO). All other data are included in the manuscript and/or *SI Appendix*.
