# Supplementary material for: Dominant and genome-wide formation of DNA:RNA hybrid G-quadruplexes in living yeast cells
Source: Proc Natl Acad Sci U S A. 2024 Oct 23;121(44):e2401099121. doi: 10.1073/pnas.2401099121 (PMC11536079; doi:10.1073/pnas.2401099121)
Supplement: Supplementary file 1 — Appendix 01 (PDF) [file pnas.2401099121.sapp.pdf]

PNAS
www.pnas.org

Supplementary Information for

Dominant and Genome-Wide Formation of DNA:RNA Hybrid G-Quadruplexes in Living Yeast Cells

Chen-xia Ren[a], Rui-fang Duan[a], Jia Wang[a], Yu-hua Hao[b], Zheng Tan[a,b,1]

[a]Shanxi Key Laboratory of Aging Mechanism Research and Translational Applications, Center for Healthy Aging, Central Laboratory, Changzhi Medical College, Changzhi 046000, Shanxi, People's Republic of China

[b]State Key Laboratory of Membrane Biology, Institute of Zoology, Chinese Academy of Sciences, Beijing 100101, People's Republic of China

[1]Address correspondence to Zheng Tan, E-mail: z.tan@ioz.ac.cn

This PDF file includes:

Supplementary text

Table S1-S2

Figures S1 to S30

SI References

Supplementary Information Text

Materials and Methods

Electrophoretic mobility shift assay (EMSA). DNA:RNA heteroduplexes were assembled as described (1) by mixing synthetic oligonucleotides, 200 nM each, in 20 mM Tris-HCl (pH 7.4) buffer containing 150 mM KCl, 1 mM EDTA, 0.4 mg/ml BSA, followed by heating to 95 °C and then slow cooling to room temperature. The DNA:RNA constructs were then incubated with 20 nM G4P at 4 °C for 5 min. The samples were resolved on 6% native polyacrylamide gel containing 50 mM KCl in 1× TBE buffer containing 50 mM KCl at 4 °C for 15 min and visualized by the FAM dye covalently attached to the 5' end of the DNA on a ChemiDoc MP imager (Bio-Rad).

Data from public repositories. The yeast chromosome sequences (sacCer3) and bed files for yeast SGD genes were downloaded from the UCSC Genome Browser (http://genome-asia.ucsc.edu). Five paired-end OK-Seq datasets (GSE115897, GSE118078, GSE139860, GSE141884, GSE173065) of Okazaki fragments (OKFs) in either bed or bedpe format (2-6) were downloaded from the Gene Expression Omnibus database (GEO, https://www.ncbi.nlm.nih.gov/geo/) hosted on the National Center for Biotechnology Information (NCBI) website. Data in bedpe format were converted to bed format using a custom-modified Perl script that we adapted from bedpe2bed.pl, which is available at https://github.com/srinivasramachandran/sam2bed/blob/main/bedpe2bed.pl. Two G4access datasets (SRR16700977 and SRR16700978) in interleaved paired-end fastq format were downloaded from the GEO under an accession number GSE187007.

Identification of PQS and control motifs in genome. A regular expression, G{3,}(.{1,7}?G{3,}){0,}, was used to search the chromosome sequences for PQSs containing one or more $G_{\geq 3}$ tracts as described (7). The number of G-tracts in the PQSs found was determined by the regular expression G{3,4}. For example, a G_7 PQS would be identified as having two G_3 tracts. Based on such analyses, we organized the resulting PQSs into multiple bed files based on the number of G-tracts. Each bed file was then divided into two separate bed files; one containing PQSs on the forward strand and the other containing PQSs on the reverse strand.

A similar approach was used to identify PQSs containing one or more $G_{\geq 2}$ tracts with the regular expression G{2,}(.{1,7}?G{2,}){0,}. Subsequently, the PQSs identified were searched with the regular expression G{3,} to remove motifs containing any $G_{\geq 3}$ tract, leaving only those that could only form hG4s or dG4s of only two G-tetrads. DNA motifs containing one or more $A_{\geq 3}$ tracts were identified using the regular expression A{3,}(.{1,7}?A{3,}){0,}. Motifs containing one or more GNG tracts, where N is any non-G nucleotide, were identified using the regular expression [^G]{7}G[^G]G(.{1,7}? G[^G]G){0,} [^G]{7}.

Identification of Orphan PQSs. PQS bed files were processed by the Bedtools merge software (8) with the arguments "-d n -c 4,5,6 -o count,collapse,distinct", where the "-d n" represents the maximum distance "n" between PQSs to be merged. PQSs that were not merged and flagged with a count of one were collected as "orphan PQSs".

Survey of PQSs in genes. The gene bed files were extended by 120 bp on the upstream side to include the promoters (9, 10) using the Bedtools slop software (8). The number of PQSs overlapping with the extended gene regions by at least one bp was counted using the Bedtools map software (8).

Distribution of Okazaki fragments (OKFs) across PQSs. The multiple bed files of OKFs in each published study were downloaded from GEO and merged into a single bed file, and then converted to bedgraph format using the Bedtools genomeCoverageBed software (8) to represent the full length (-bg -strand +/-), 3' (-bg -3 -strand +/-), or 5' (-bg -5 -strand +/-) ends of the OKFs on the plus and minus strand, respectively. The resulting bedgraph files were then converted to bigwig format using the Bedtools bedGraphToBigWig software (8) with a bin size of 10 nucleotides (nts), unless specified otherwise. Each PQS bed files was split into two according to the strand orientation (i.e. plus and minus) and then used as

region files for OKF profiling. The distribution of OKFs and their 3' or 5' ends over the 3'-end of PQSs was profiled using the computeMatrix and plotProfile tools of the Deeptools software (11), along with the corresponding bigwig and PQS bed files.

To profile the 5' end of the OKFs whose 3'-end was within ±20 nts of the 3'-end of a PQS, a new PQS bed file was created from the original PQS bed file to represent the PQS 3' ±20 nts regions. A new OKF bed file was also created by copying columns 2 and 3 to columns 7 and 8 of the original OKF bed file and then modifying columns 2 and 3 to represent the OKF 3'-ends. The overlap of regions between the two bed files was determined using the intersect command of the Bedtools software (28) to remove the OKFs in the OKF bed file whose 3'-end did not overlap with any regions in the PQS bed file. Columns 7 and 8 of the OKF bed file were then restored to columns 2 and 3, respectively. The resulting OKF bed file was then processed to profile the distribution of the 5'- and 3'-ends of the OKFs at the PQS 3'-ends as described in the previous section.

Plasmid construction for G4 probe (G4P) ChIP. The coding sequence of the G4P protein was amplified from pNLS-G4P-IRES2-EGFP (12) using a PCR primer pair of 5'-ATTAAGCTTATGCCCAAGAAGAAGC-GGAAG-3' and 5'-GTGGATCCTTACTTGTCATCGTCATCCTT-3'and inserted into the pYES2 plasmid between the Hind III and BamH I sites.

G4P ChIP-Seq. The recombinant plasmid pYES2-G4P-3xFLAG was introduced into *S. cerevisiae* strain BY4741 using the lithium acetate/PEG transformation method (13). The transformant was inoculated on SC-Ura agar medium (synthetic dropout agar medium without uracil). Colonies were collected after 72 hrs of incubation at 30 °C and then cultured in SD-Ura liquid medium (synthetic complete medium without uracil supplemented with 2% (w/v) glucose) at 30 °C to an OD_{600} of 2. Cells were then harvested by centrifugation and washed three times in SG-Ura liquid medium (synthetic complete medium without uracil supplemented with 2% (w/v) galactose). Cell pellets were resuspended in liquid SG-Ura to an OD_{600} of 0.6 and incubated at 30 °C to activate the GAL1 promoter.

After 6 hrs of G4P induction, cells were cross-linked with 1% formaldehyde and then mixed with 425-600 μm glass beads (Cat# G8080, Solarbio, China) and lysed using a bead ruptor (Bioprep-6, Allsheng, Hangzhou, China) at 6.0 m/s for 8 cycles of 30 sec on at 4 °C and 10 min off on ice. The resulting lysate was pelleted, resuspended in 600 μl of lysis buffer and then sheared using a Covaris M220 ultrasonicator for 20 min at 4-7 °C (10% duty cycle, 75 W power, 200 burst).

For DNA sequencing, 1% of the DNA fragment sample was saved as input, and the remainder was immunoprecipitated using 30 μl anti-FLAG M2 magnetic beads (Cat# M8823, Sigma-Aldrich) according to the protocol (14). Purified DNA was sequenced on an Illumina HiSeq platform (Genewiz, Suzhou, China) to generate 2×150 bp paired-end reads for input and G4P-bound DNA. Clean fastq data were then aligned to the UCSC sacCer3 genome using the Bowtie2 software and mapped to PQSs or other indicated motifs as described (12).

For sequencing RNA in hG4s, 5 μg of monoclonal DNA antibody (Cat# MA1-83116, Invitrogen) was incubated for 2 hrs at 4 °C with Protein A/G Mix Magnetic Beads (Cat# LSKMAGAG10, Millipore). After washing with dilution buffer, the beads were mixed with DNA fragments and immunoprecipitation (IP) was performed as described (14). The resulting elution, without cross-linking reversal, was split into two halves, one half incubated with 20 μl mouse IgG anti-FLAG M2 magnetic beads for G4 pull-down and the other half incubated with 20 μl mouse IgG magnetic beads (Cat#5873, Cell Signaling Technology) for non-specific control. Immunoprecipitation was conducted as described (14). The elution was subjected to cross-linking reversal and then treated with DNase I and Proteinase K, respectively. The RNA was purified using an RNA MinElute column (Cat# 74204, Qiagen) and was further treated with DNase I. The remaining RNA was then purified using the RNA MinElute column. RNA library was prepared and strand-specific RNA-Seq was performed on an Illumina HiSeq platform (Genewiz, Suzhou, China).

To determine the presence of PQSs in hG4 RNAs, the R1 reads of the RNA-Seq data in fastq format were first processed using the fastp software with the following options: "--length_required 5 --length_limit 55 --dedup", with the remaining options left as defaults. The resulting fastq data was then aligned to the

UCSC sacCer3 genome using the Hisat2 software with the options “-k 1 --fast --no-softclip --no-unal”. The samtools view software was then used with the options “-q 20 -F 0x100” to filter for the aligned RNA reads, discarding unaligned reads and ensuring that each read was only reported once, if it could be aligned to multiple positions in the genome. The resulting bam files were converted to fasta format using the Samtools view and awk commands. For reads aligned in the reverse direction, their sequences were reverse-complemented using the Seqkit seq software. Finally, the PQS motifs in the fasta files were identified as described previously (15).

Distribution of G4P across PQSs. Distribution of G4P binding across regions of interest was calculated as described (12).

Distribution of G4access signals across PQSs. Fastq files (accessions SRR16700977 and SRR16700978) (16) were downloaded from NCBI and aligned to the yeast genome (sacCer3) using the Bowtie2 software (17) with the sensitive-local preset and the parameters --no-unal, --no-discordant, --no-mixed. Mapped reads were written to bam files after being filtered by the Samtools view (18) to remove poor quality alignments with the parameter -q 30 and by the Samtools rmdup to remove duplicates. The resulting bam files were processed using the Deeptools bamCoverage tool (11) to generate bigwig files normalized to RPKM with a bin size of 10 nts. Profiles of reads were generated from the bigwig files using the computeMatrix tool, followed by the plotProile tool of the Deeptools software (11), with PQS region bed files. The final profile was the average of two replicates.

Table S1. DNA and RNA oligomers used in DNA polymerase stop assay (Figure 2).

Panel	Lane	Sequences (top:5'-3')
B	1	ctctcttaacGccGaGGGGaaGGGGaGGGGtgtatacactgaagcgatcgtgatac<br>ttcgctagcactatg-FAM-5'
	2	ctctcttaacGGGGaGGGGaaGGGGaGGGGtgtatacactgaagcgatcgtgatac<br>ttcgctagcactatg-FAM-5'
	3	Tgtatacactgaagcgatcgtgatac<br>ttcgctagcactatg-FAM-5'
C	2	ctctcttaacGGGttGGGttGGGttGGGttGGGttGGGttGGGtgtatacactgtttatcgaacggtg<br>caaatagcttgccac-FAM-5'
D	1	atgtatacactgaagcgatcgtgatactccacaataagataaacttatattc<br>ctatttgaatataag-FAM-5'
	2	aGGGatgtatacactgaagcgatcgtgatactccacaataagataaacttatattc<br>ctatttgaatataag-FAM-5'
	3	ctcaacaactaactcagcgactgaGGGatgtatacactgaagcgatcgtgatactccacaataagataaacttatattc<br>gaguuguugauugagucgcugacaGGGaGGGaGGGa ctatttgaatataag-FAM-5'
	4	ctcaacaactaactcagcgactgaGGGaGGGatgtatacactgaagcgatcgtgatactccacaataagataaacttatattc<br>gaguuguugauugagucgcugacaGGGaGGGa ctatttgaatataag-FAM-5'
	5	ctcaacaactaactcagcgactgaGGGaGGGaGGGatgtatacactgaagcgatcgtgatactccacaataagataaacttatattc<br>gaguuguugauugagucgcugacaGGGa ctatttgaatataag-FAM-5'
	6	ctcaacaactaactcagcgactgaGGGaGGGaGGGaGGGatgtatacactgaagcgatcgtgatactccacaataagataaacttatattc<br>gaguuguugauugagucgcugacaGUGaGUGaGUGa ctatttgaatataag-FAM-5'
	7	ctcaacaactaactcagcgactgaGTGaGTGaGTGaGTGatgtatacactgaagcgatcgtgatactccacaataagataaacttatattc<br>gaguuguugauugagucgcugacaGUGaGUGaGUGa ctatttgaatataag-FAM-5'
	8	ctcaacaactaactcagcgactgaGGGaGGGaGGGaGGGatgtatacactgaagcgatcgtgatactccacaataagataaacttatattc<br>gaguuguugauugagucgcugacaGGGaGGGaGGGa ctatttgaatataag-FAM-5'

* Hybridization regions are underlined.

Table S2. DNA and RNA oligomers used in EMSA (Figure S7).

Lane	Sequences (top:5'-3')
1,2	FAM-5'-ctcaacaactaactcagcgactgaGGGa<br>gaguuguugauugagucgcugacaGGGaGGGaGGGa
3,4	FAM-5'-ctcaacaactaactcagcgactgaGGGaGGGa<br>gaguuguugauugagucgcugacaGGGaGGGa
5,6	FAM-5'-ctcaacaactaactcagcgactgaGGGaGGGaGGGa<br>gaguuguugauugagucgcugacaGGGa
7,8	FAM-5'-ctcaacaactaactcagcgactgaGGGaGGGaGGGaGGGa<br>gaguuguugauugagucgcugacaGUGaGUGaGUGa
9,10	FAM-5'-ctcaacaactaactcagcgactgaGTGaGTGaGTGaGTGa<br>gaguuguugauugagucgcugacaGUGaGUGaGUGa
11,12	FAM-5'-ctcaacaactaactcagcgactgaGGGaGGGaGGGaGGGa<br>gaguuguugauugagucgcugacaGGGaGGGaGGGa

* Hybridization regions are underlined.

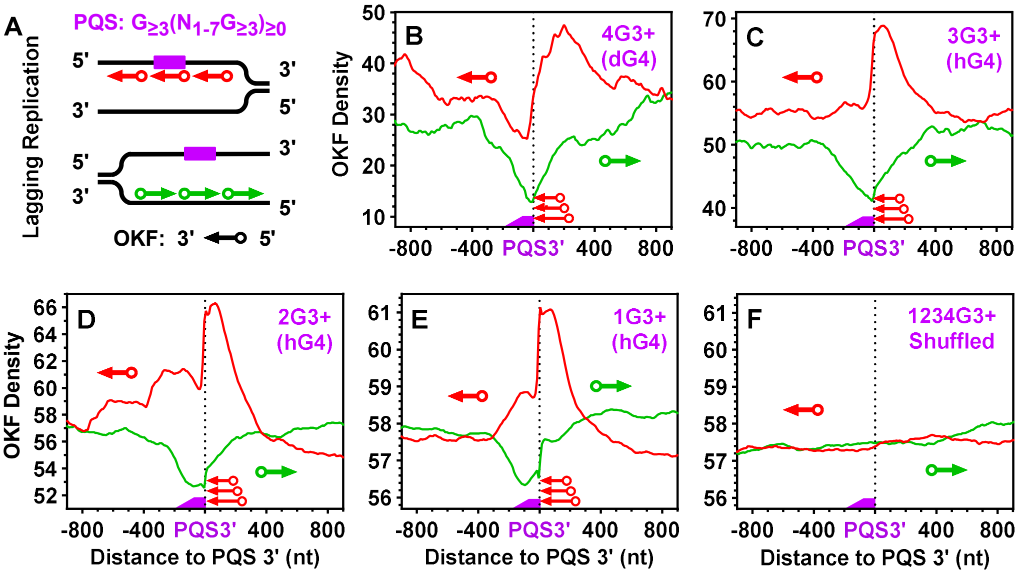

Figure S1. Distribution of OKFs at the 3’ end of PQSs with 1 to 4 or more $G_{\geq 3}$ tracts that could form either dG4s or hG4s of three or more G-tetrads. Same as in Figure 3, except original OK-Seq data from GSE118078.

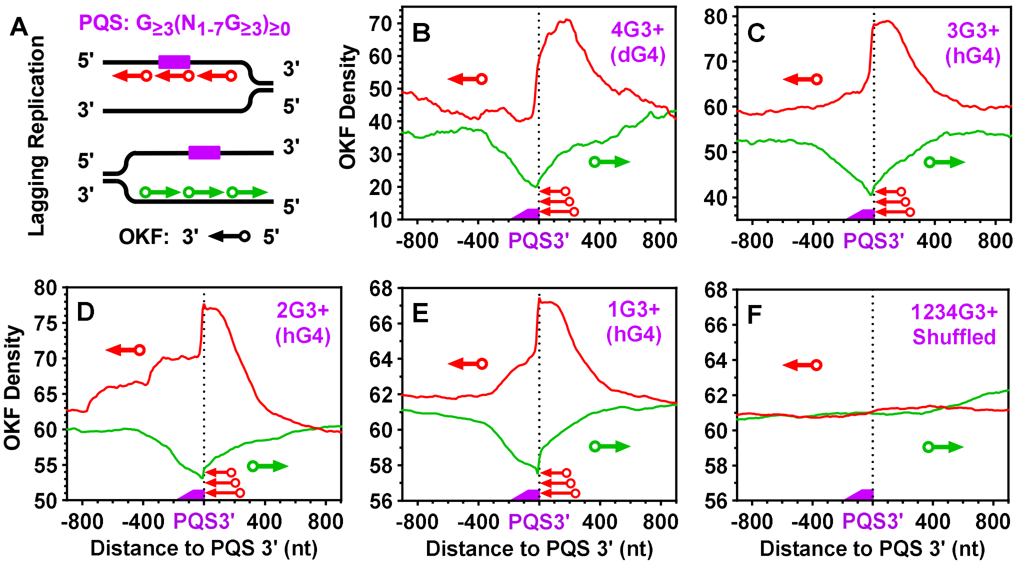

Figure S2. Distribution of OKFs at the 3' end of PQSs with 1 to 4 or more $G_{\geq 3}$ tracts that could form either dG4s or hG4s of three or more G-tetrads. Same as in Figure 3, except original OK-Seq data from GSE139860.

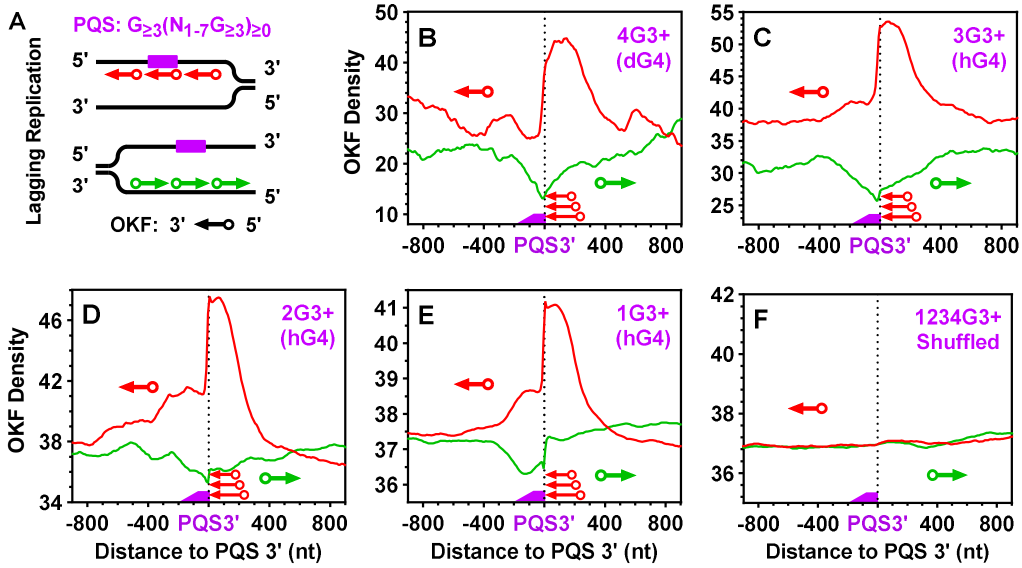

Figure S3. Distribution of OKFs at the 3' end of PQSs with 1 to 4 or more $G_{\geq 3}$ tracts that could form either dG4s or hG4s of three or more G-tetrads. Same as in Figure 3, except original OK-Seq data from GSE141884.

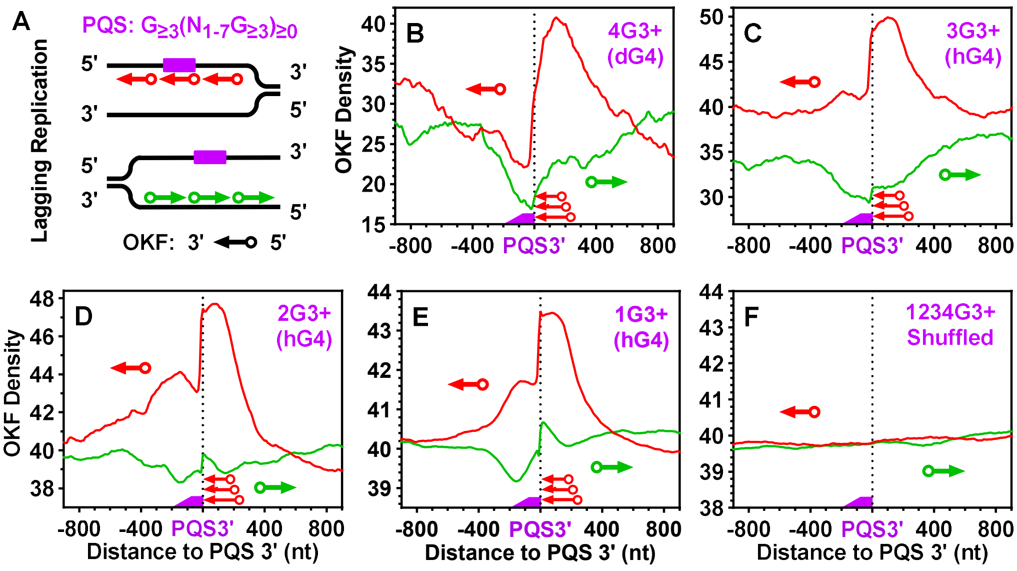

Figure S4. Distribution of OKFs at the 3' end of PQSs with 1 to 4 or more $G_{\geq 3}$ tracts that could form either dG4s or hG4s of three or more G-tetrads. Same as in Figure 3, except original OK-Seq data from GSE173065.

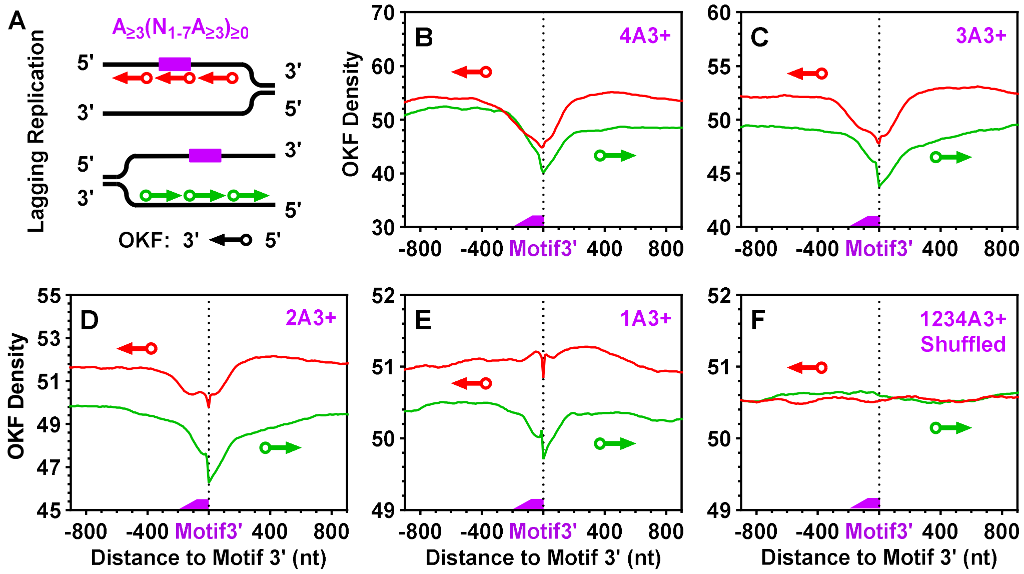

Figure S5. Distribution of OKFs at the 3' end of A-rich motifs containing 1 to 4 or more $A_{\geq3}$ tracts that are unable to form G4. Same as in Figure 3, except that the PQSs were replaced by the indicated motifs.

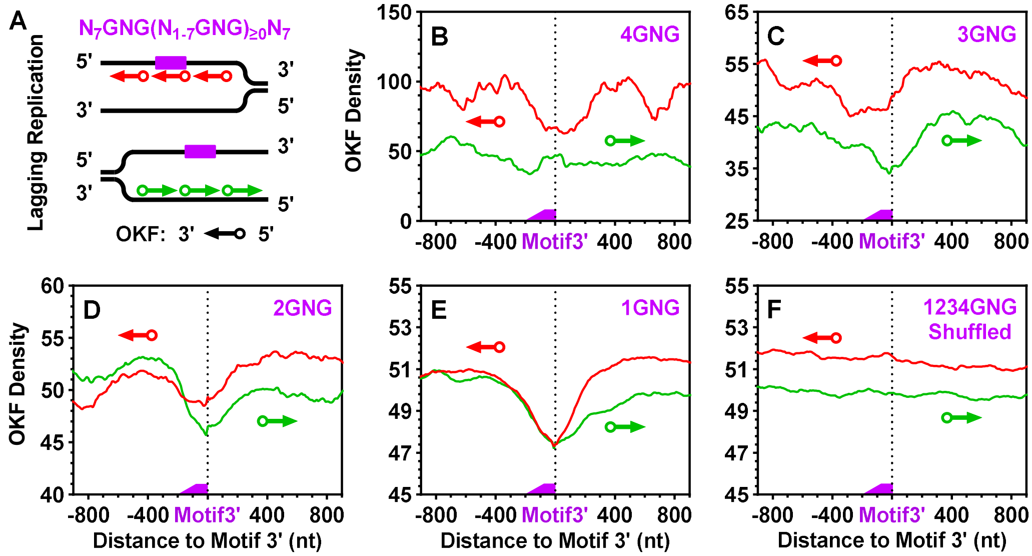

Figure S6. Distribution of OKFs at the 3' end of motifs containing 1 to 4 or more GNG tracts that are unable to form G4. Same as in Figure 3, except that the PQSs were replaced by the indicated motifs. N represents any nucleotide other than G.

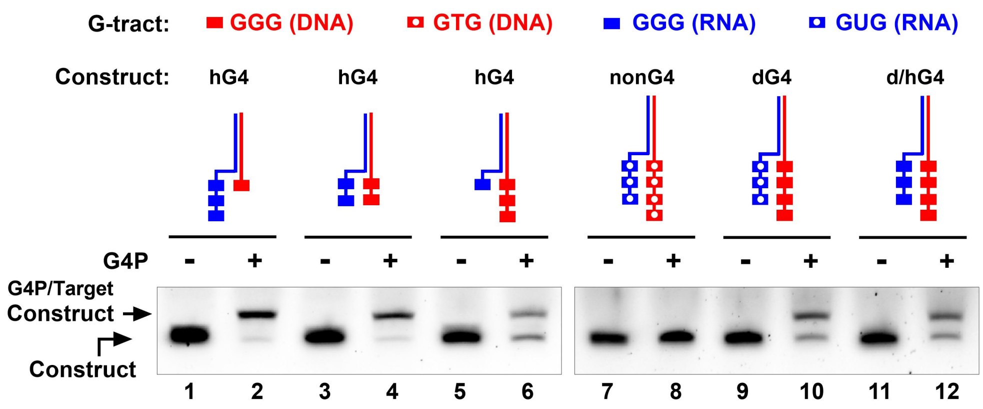

Figure S7. G4P binds hG4s with different DNA:RNA G-tract ratios (lanes 1-6), dG4 (lanes 9-10), and d/hG4 (lanes 11-12) as determined by electrophoretic mobility shift assay (EMSA). Constructs were made by annealing a 5'-FAM-labeled DNA (red line) and an RNA (blue line) carrying the indicated G-tracts or mutants. The construct in lanes 7-8 contained a single-stranded DNA and RNA and a DNA:RNA heteroduplex (R-loop core) component as controls.

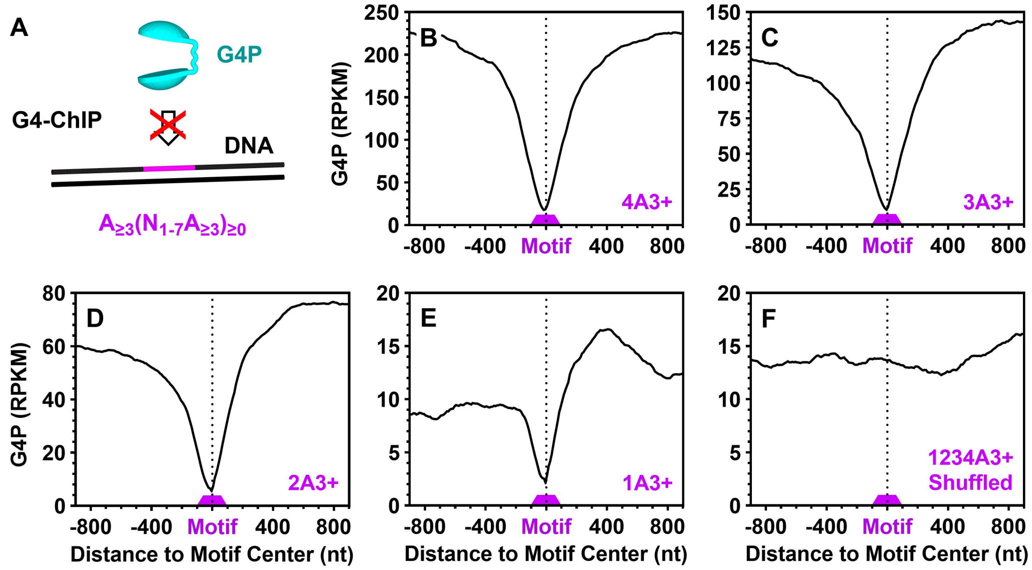

Figure S8. Binding profiles of G4P at A-rich motifs containing 1 to 4 or more $A_{\geq 3}$ tracts that are unable to form G4. Same as in Figure 4, except that the PQSs were replaced by the indicated motifs.

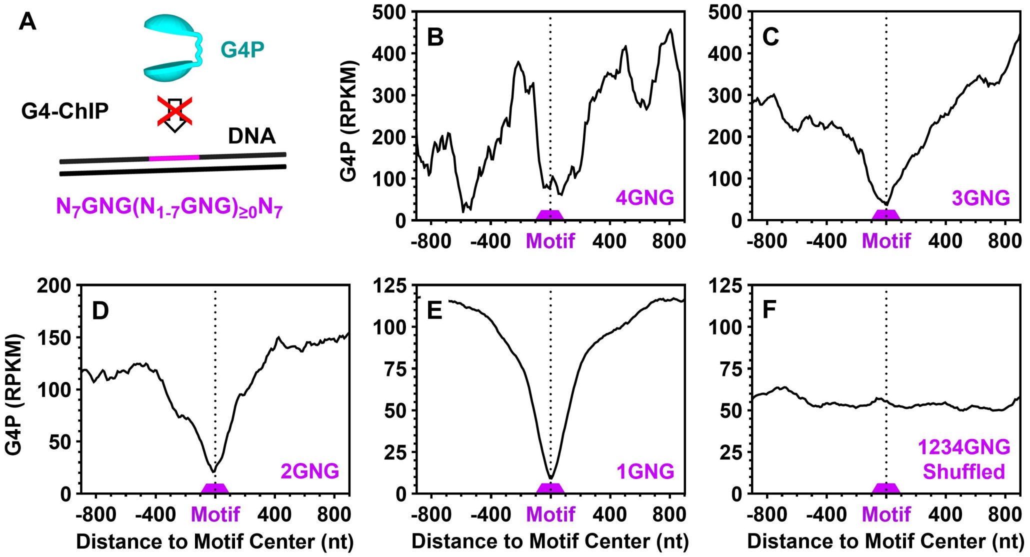

Figure S9. Binding profiles of G4P at motifs containing 1 to 4 or more GNG tracts that are unable to form G4. Same as in Figure 4, except that the PQSs were replaced by the indicated motifs. N represents any nucleotide other than G.

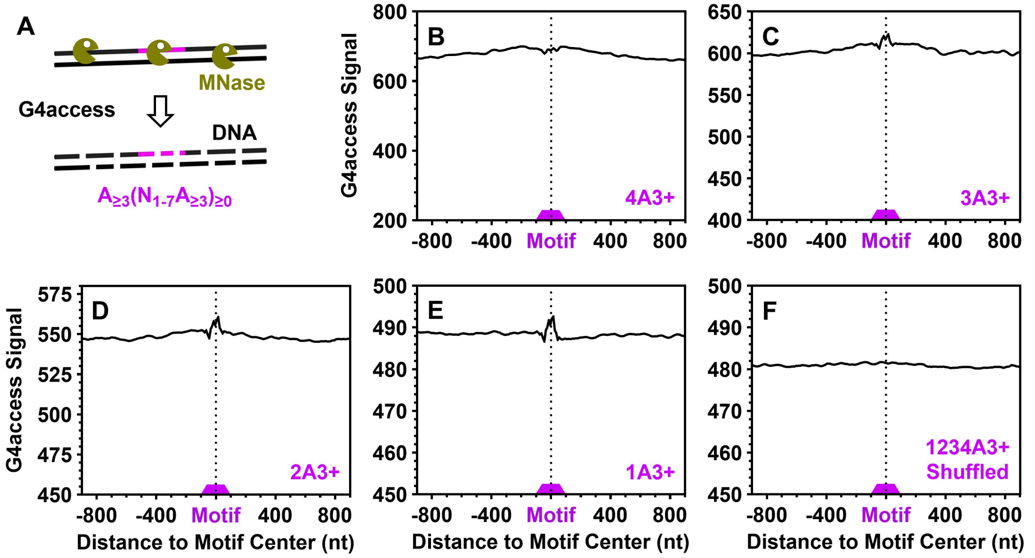

Figure S10. Distribution of G4access signals at A-rich motifs containing 1 to 4 or more $A_{\geq3}$ tracts that are unable to form G4. Same as in Figure 5, except that the PQSs were replaced by the indicated motifs.

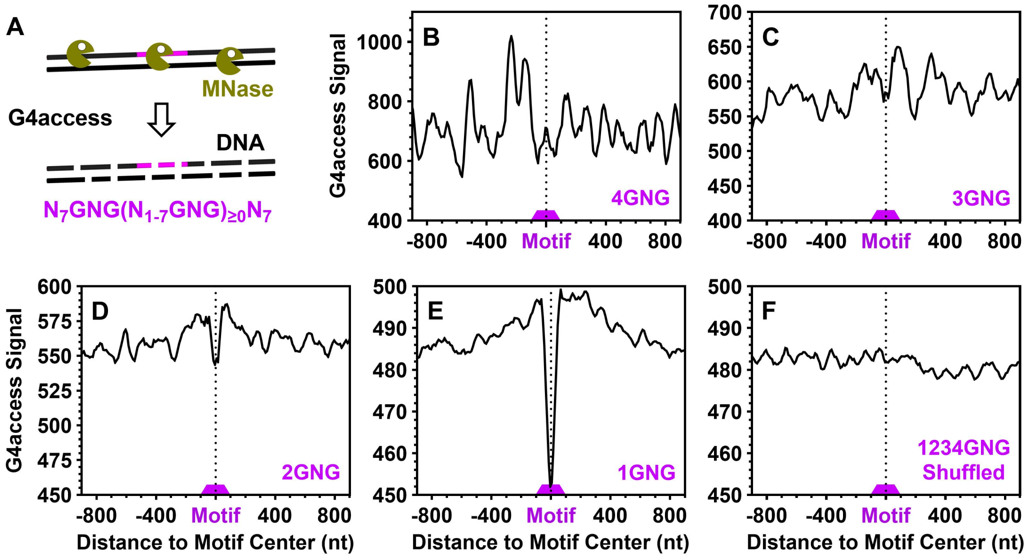

Figure S11. Distribution of G4access signals at motifs containing 1 to 4 or more GNG tracts that are unable to form G4. Same as in Figure 5, except that the PQSs were replaced by the indicated motifs. N represents any nucleotide other than G.

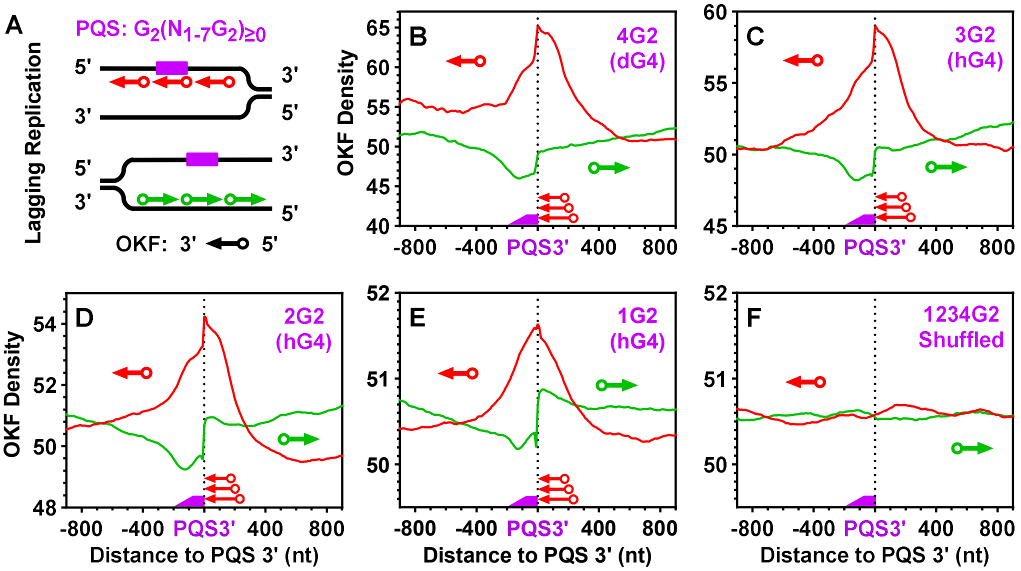

Figure S12. Distribution of OKFs at the 3' end of PQSs with 1 to 4 or more GG tracts that could form G4s of only two G-tetrads. (A) Scheme of profiling on the PQS-bearing strand and the strand opposite to a PQS. (B-E) Distribution at PQSs capable of forming (B) dG4s or (C-E) hG4s. (F) Distribution at randomly shuffled PQSs.

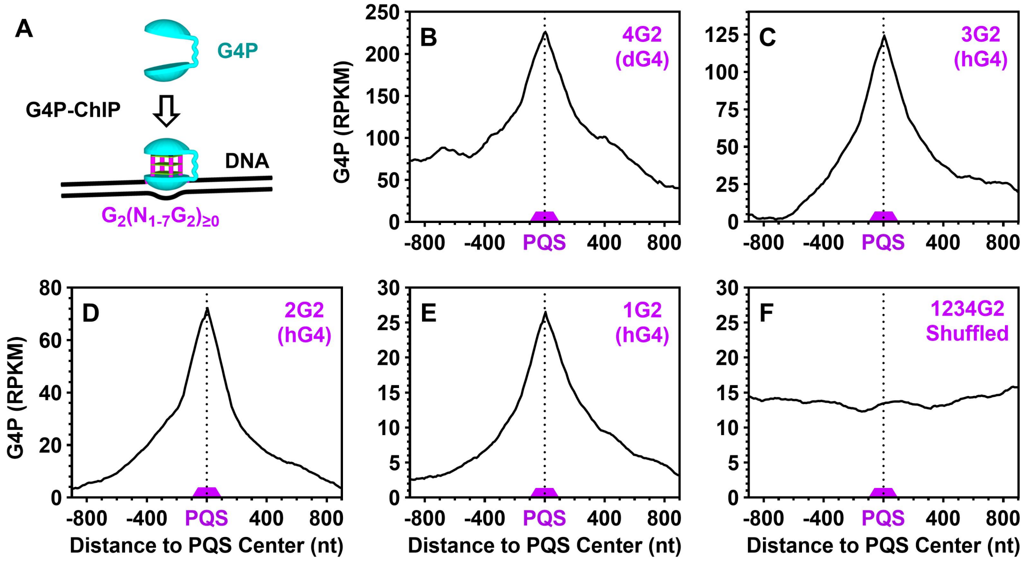

Figure S13. Binding profiles of G4P at PQSs with 1 to 4 or more GG tracts that could form G4s of only two G-tetrads. (A) Scheme of G4 detection by G4P ChIP-Seq. (B-E) Enrichment of G4P at PQSs capable of forming (B) dG4s or (C-E) hG4s. (F) Distribution of G4P at randomly shuffled PQSs.

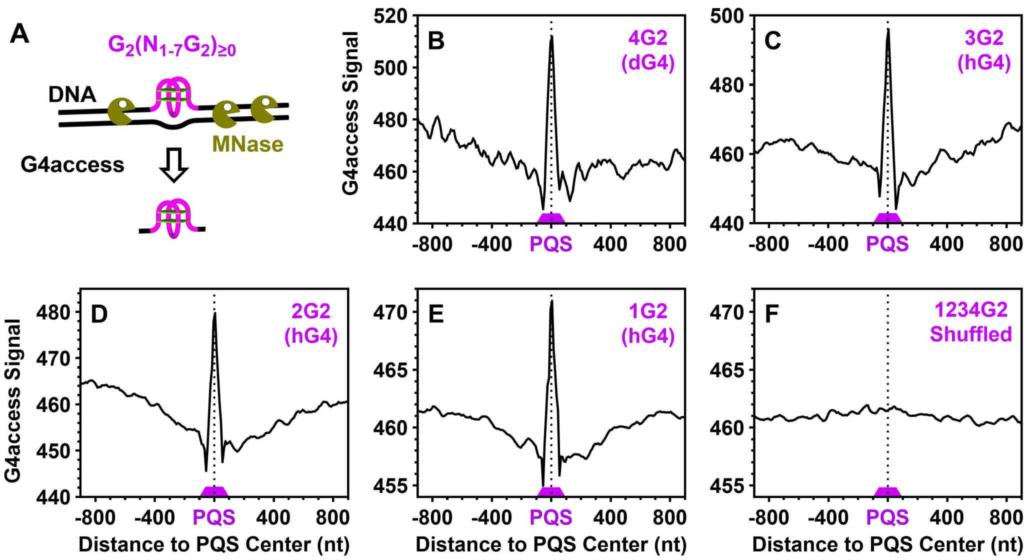

Figure S14. Distribution of G4access signals at PQSs with 1 to 4 or more GG tracts that could form G4s of only two G-tetrads. (A) Scheme of G4 detection by G4access. (B-E) Enrichment at PQSs capable of forming (B) dG4s or (C-E) hG4s. (F) Distribution at randomly shuffled PQSs.

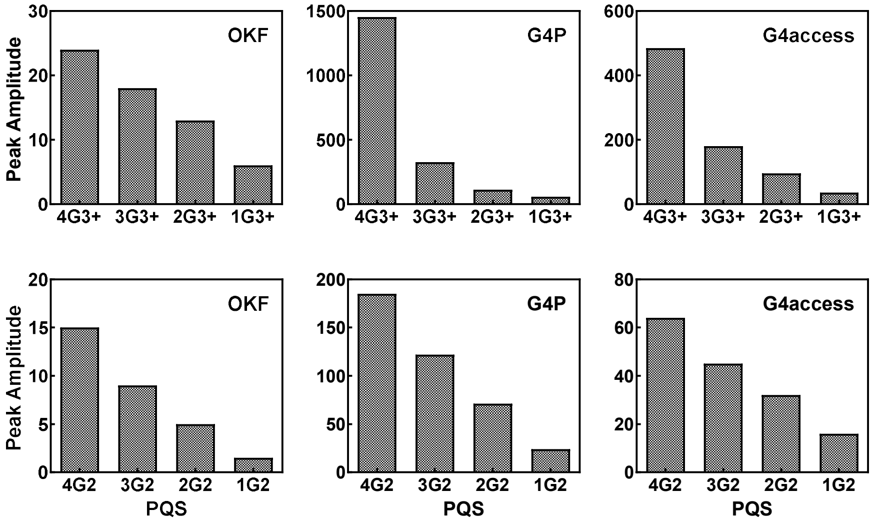

Figure S15. Dependence of G4 detection signals on the number of G-tracts of PQS. Peak amplitude estimated from Figure 3,Figure **4**,Figure **5**,Figure **S12**,Figure **S13**, andFigure **S14**, respectively.

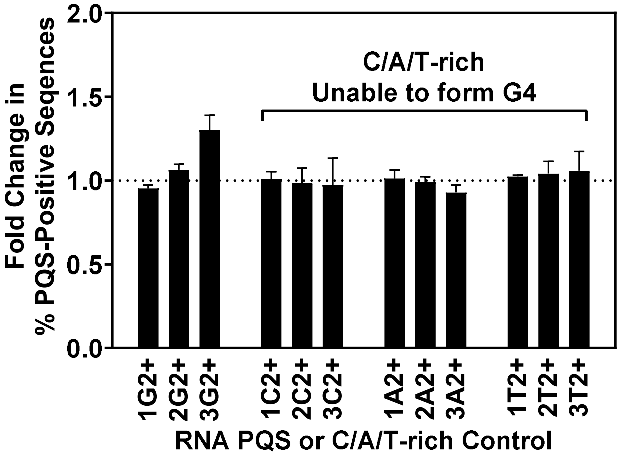

Figure S16. Detection of RNA G-tracts in hG4s with two or more G-tetrads. Same as in Figure 6B except for the size of the motif tracts.

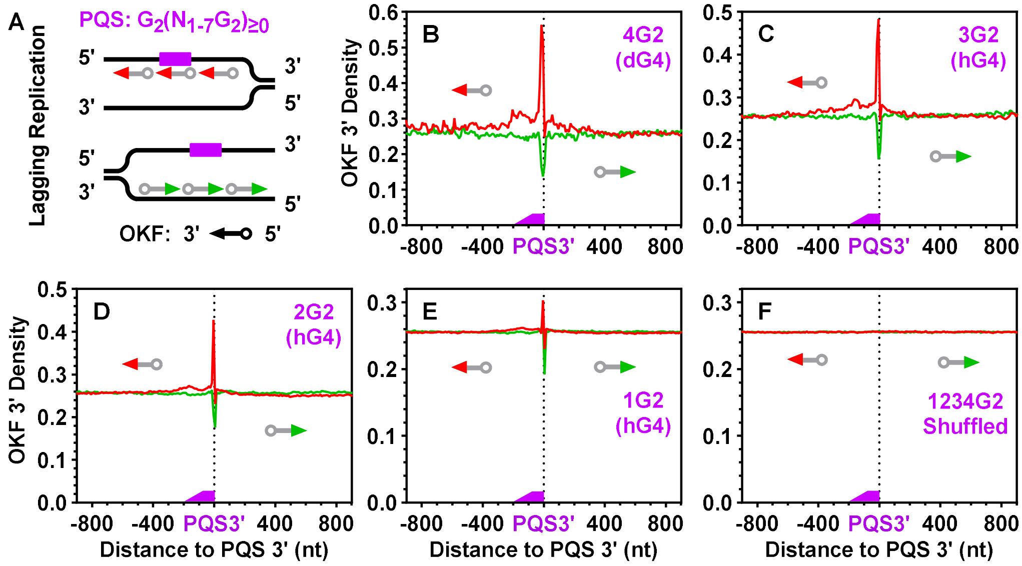

Figure S17. Distribution of OKF 3' ends at the 3' end of PQSs with 1 to 4 or more GG tracts that could form G4s of only two G-tetrads. (A) Scheme of profiling on PQS-bearing strand and strand opposite to PQS. (B-E) Distribution at PQSs capable of forming (B) dG4s or (C-E) hG4s. (F) Distribution at randomly shuffled PQSs.

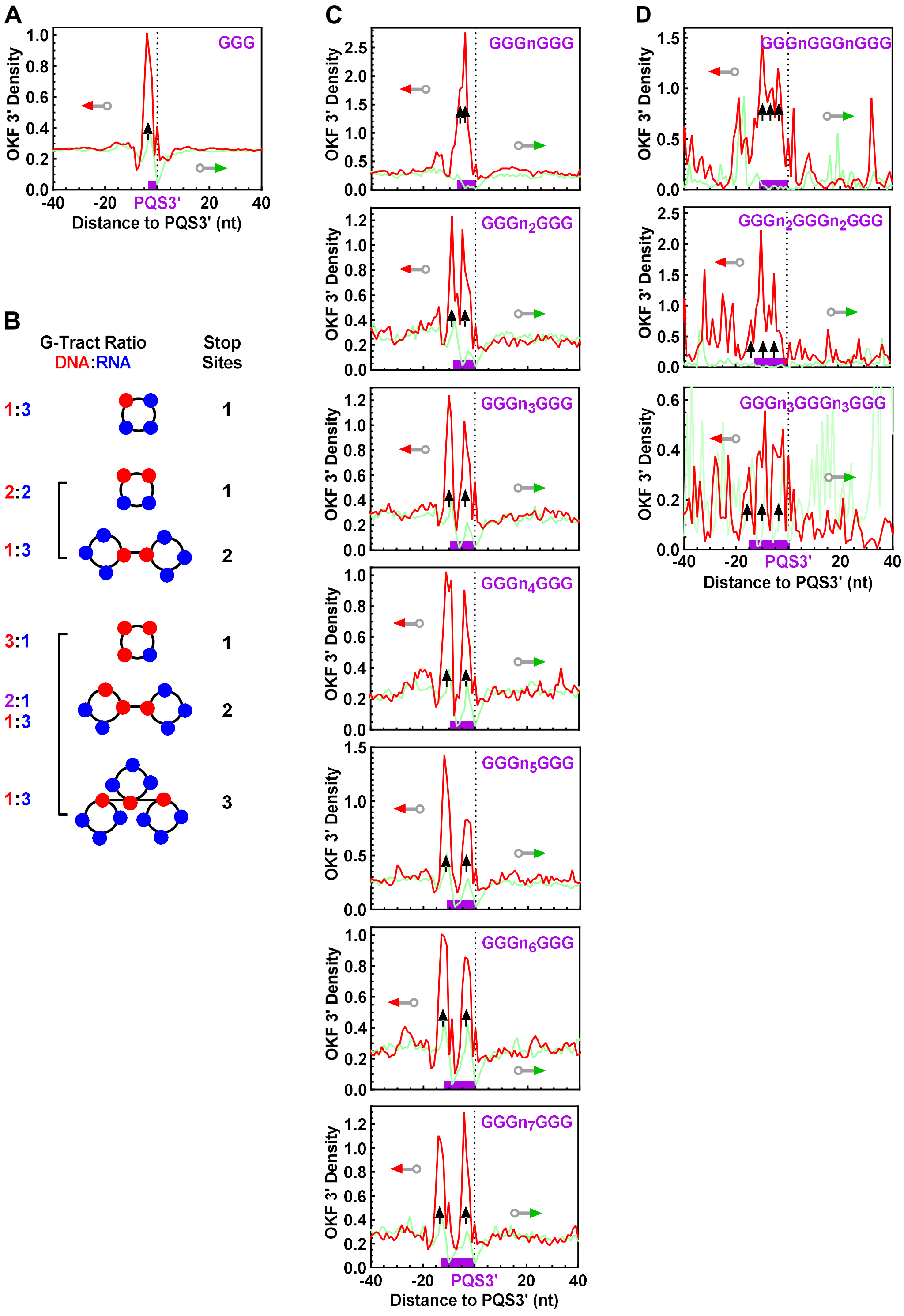

Figure S18. Distribution of OKF 3' ends at the 3' end of PQSs with (A) one, (C) two, or (D) three GGG tracts. (B) Examples of combinations of DNA and RNA G-tracts in hG4 formation. "n" denotes any nucleotide, but not G if adjacent to G. Bin size: 1 nt.

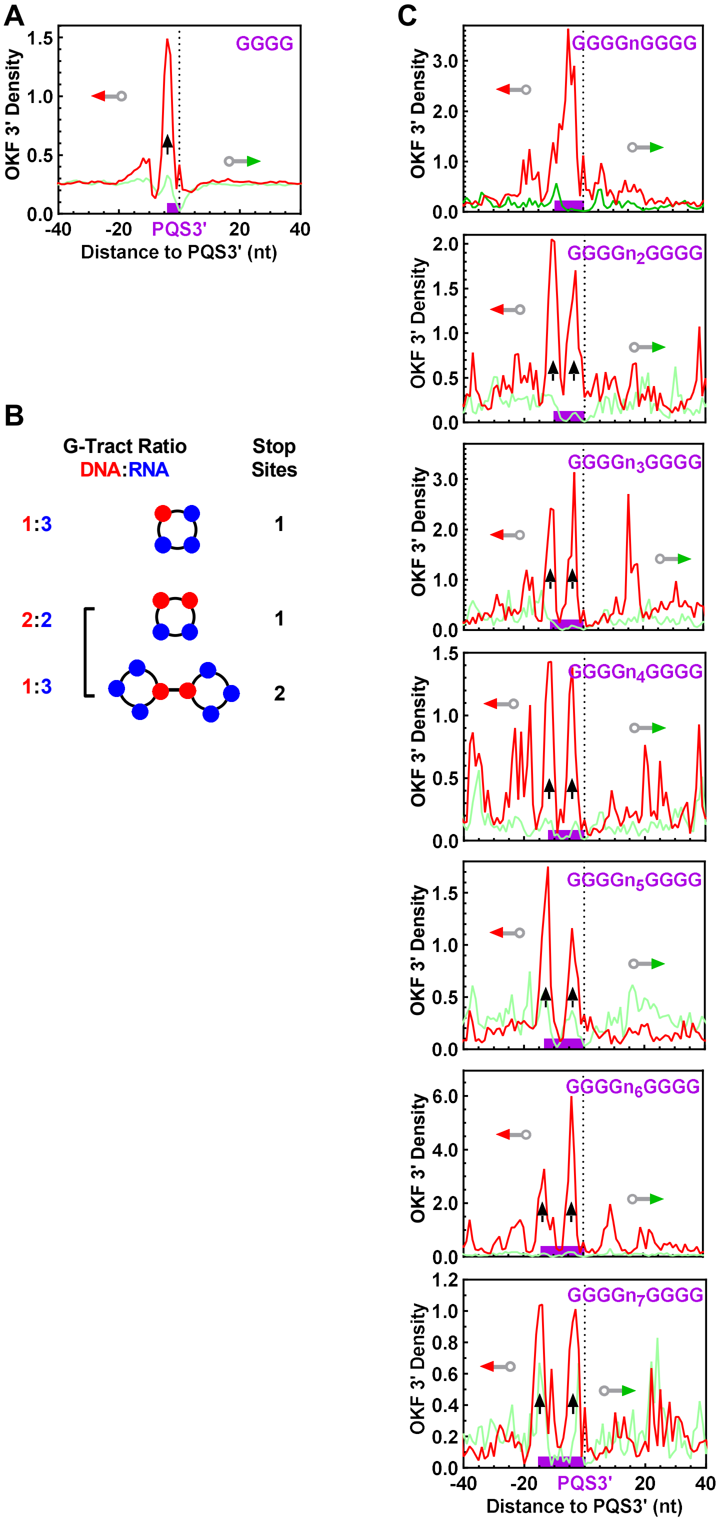

Figure S19. Distribution of OKF 3' ends at the 3' end of PQSs with (A) one or (C) two GGGG tracts. (B) Examples of combinations of DNA and RNA G-tracts in hG4 formation. "n" denotes any nucleotide, but not G if adjacent to G. Bin size: 1 nt.

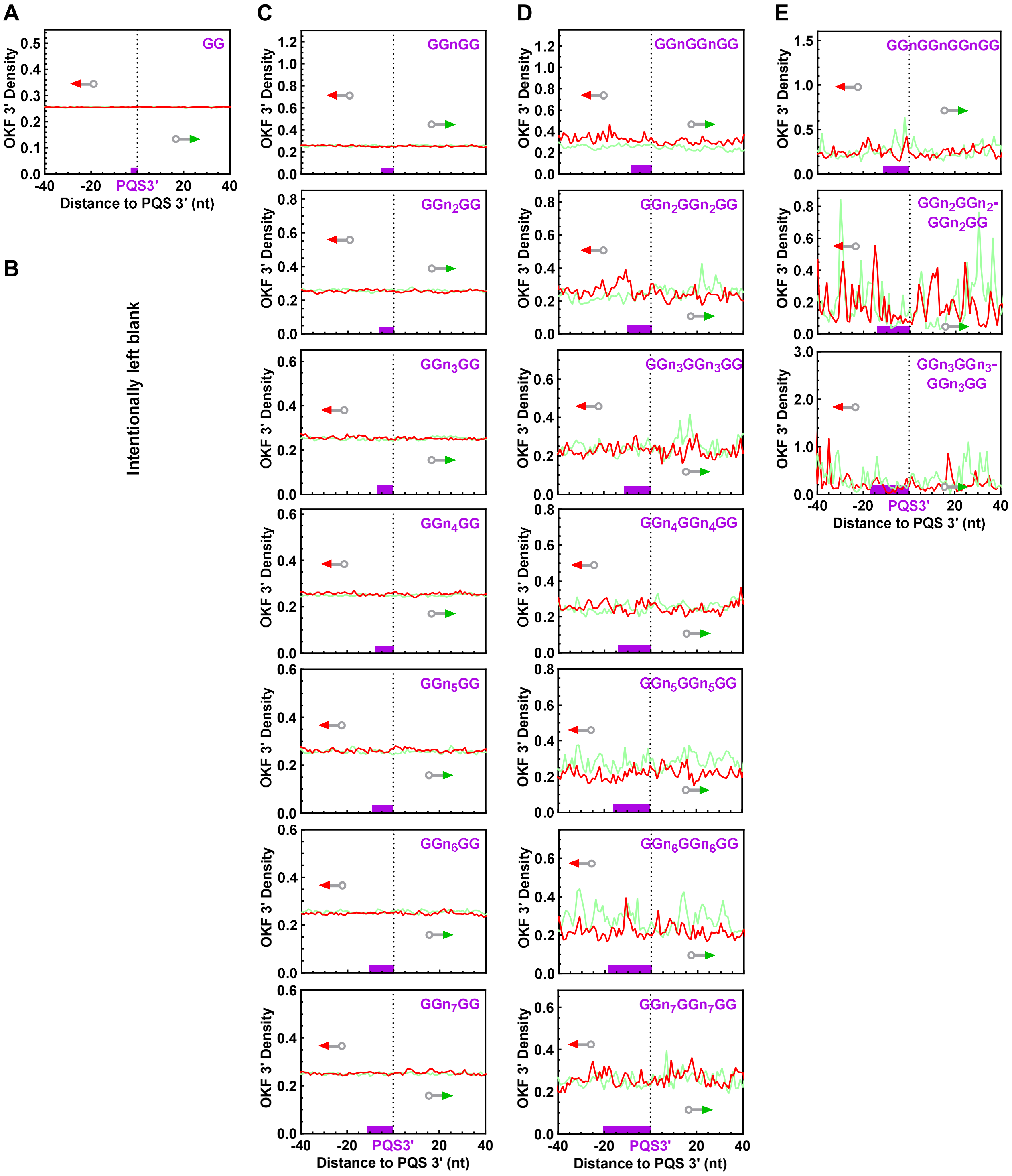

Figure S20. Distribution of OKF 3’ ends at randomly shuffled PQSs with (A) one, (C) two, (D) three, or (E) four GG tracts. “n” denotes any nucleotide, but not G if adjacent to G. Same as in Figure 9, except that the coordinates of the PQSs were shuffled to random locations. Bin size: 1 nt.

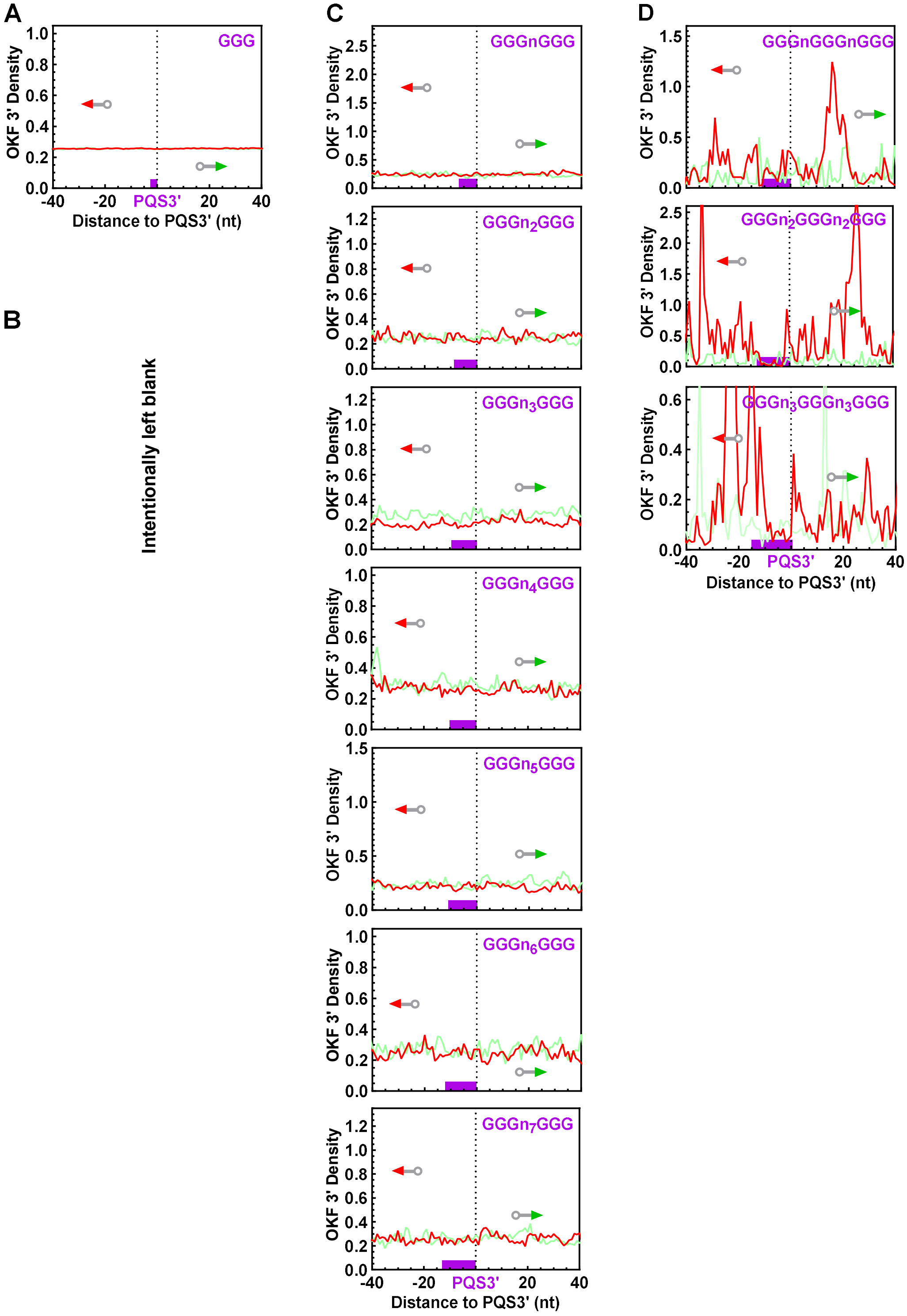

Figure S21. Distribution of OKF 3' ends at randomly shuffled PQSs with (A) one, (C) two, and (D) three GGG tracts. "n" denotes any nucleotide, but not G if adjacent to G. Same as in Figure S18, except that the coordinates of the PQSs were shuffled to random locations. Bin size: 1 nt.

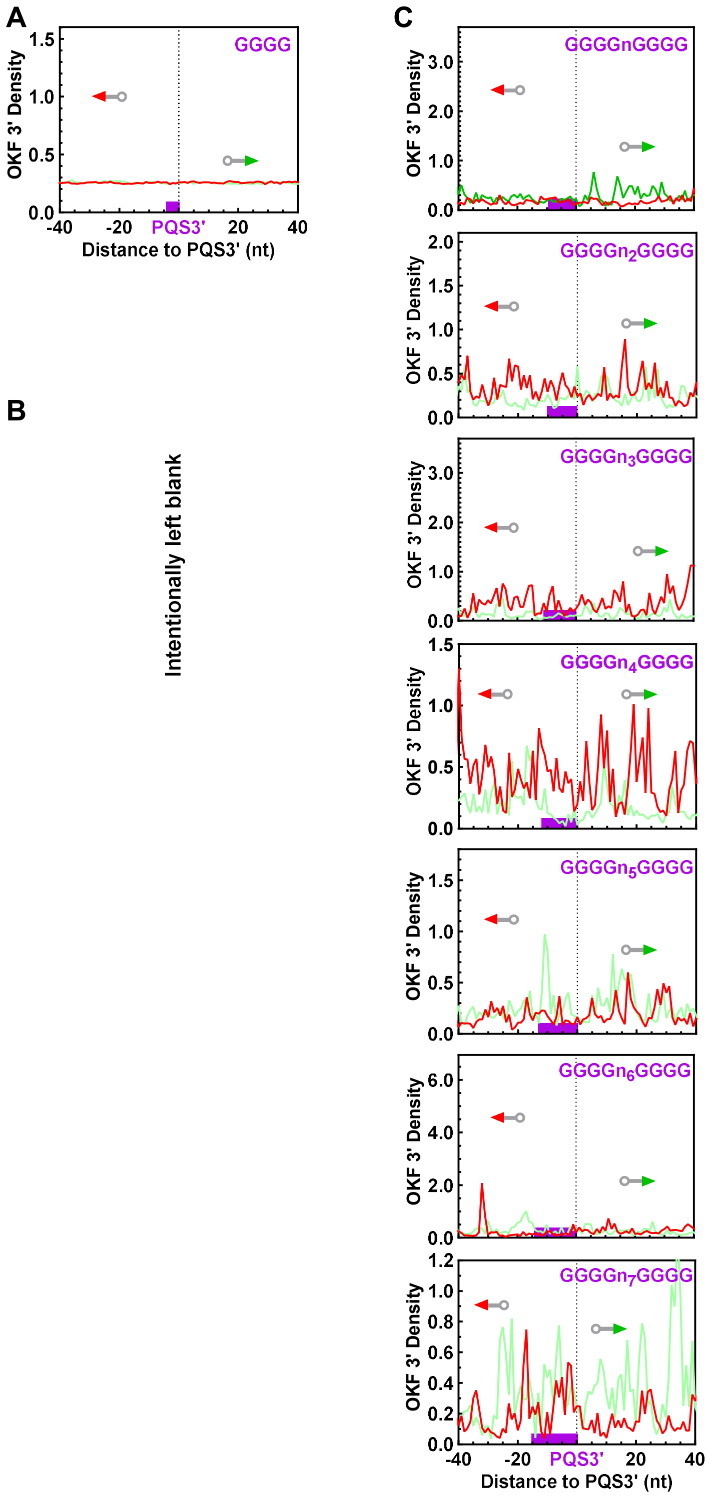

Figure S22. Distribution of OKF 3' ends at randomly shuffled PQSs with (A) one, (C) two GGGG tracts. "n" denotes any nucleotide, but not G if adjacent to G. Same as in Figure S19, except that the coordinates of the PQSs were shuffled to random locations. Bin size: 1 nt.

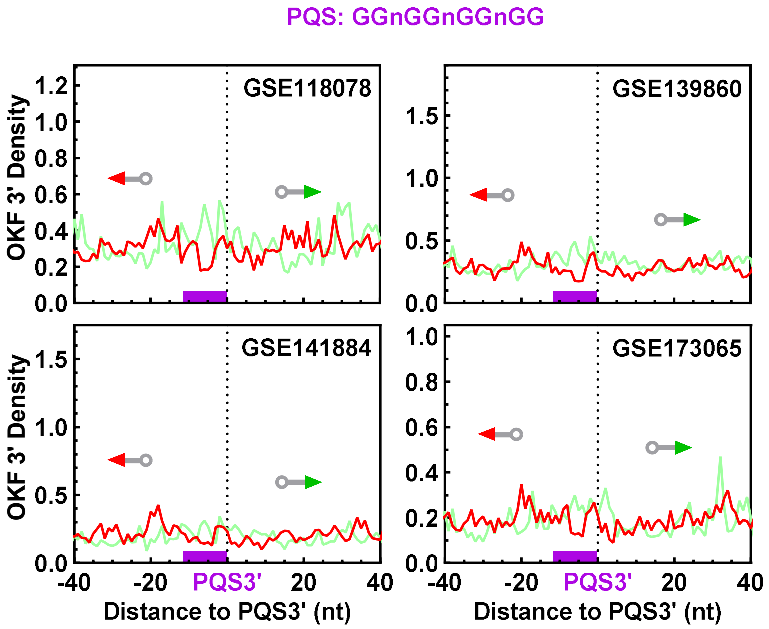

Figure S23. Distribution of OKF 3’ ends at randomly shuffled PQSs with four GG tracts and 1-nt loops. “n” denotes any nucleotide other than G. Same as in Figure 10, except that the coordinates of the PQSs were shuffled to random locations. Bin size: 1 nt.

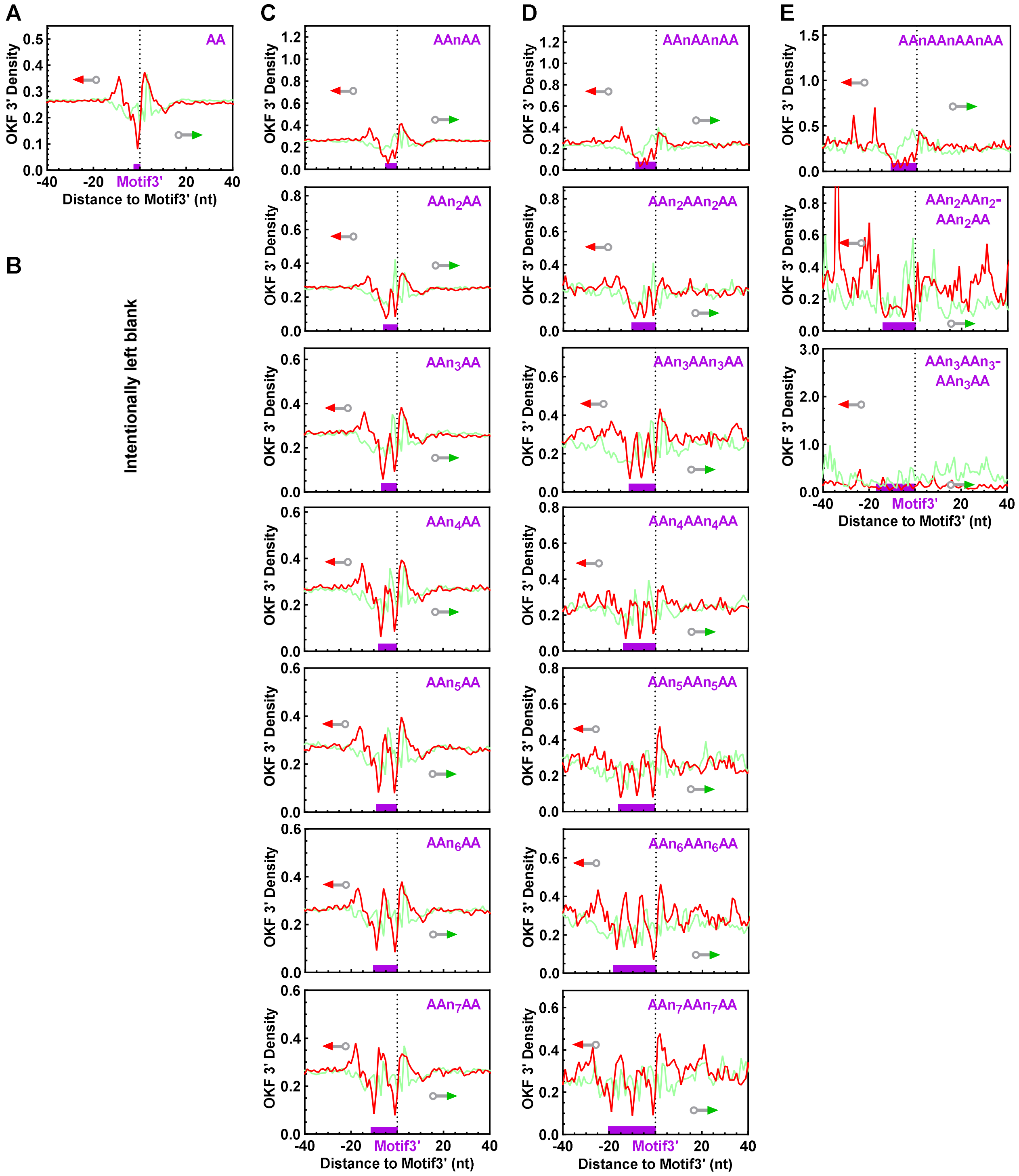

Figure S24. Distribution of OKF 3' ends at the 3' end of A-rich motifs containing (A) one, (C) two, (D) three, or (E) four AA tracts. "n" denotes any nucleotide, but not A if adjacent to A. Same as in Figure 9, except profiled across the indicated A-rich motifs. Bin size: 1 nt.

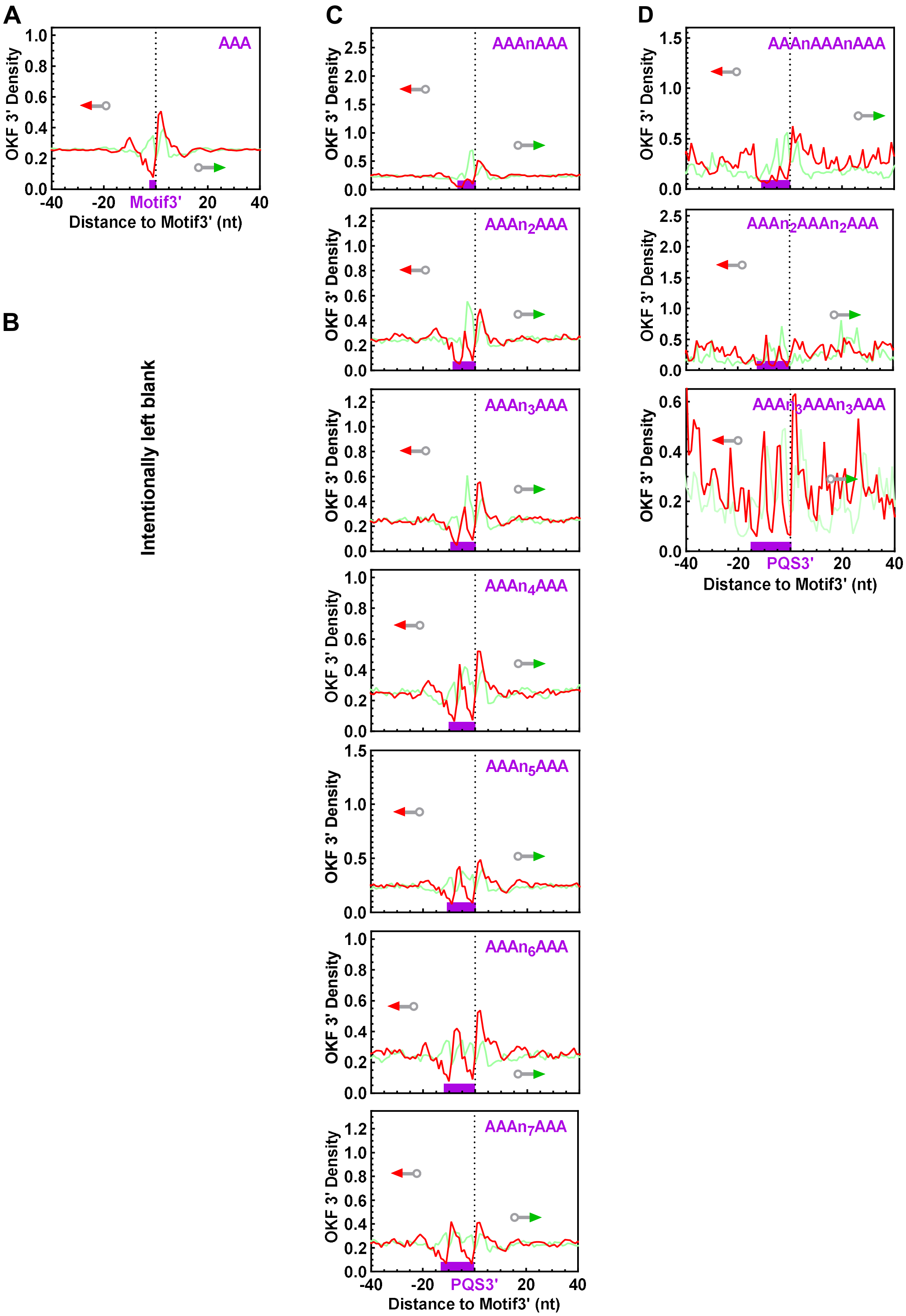

Figure S25. Distribution of OKF 3' ends at the 3' end of A-rich motifs containing(A) one, (C) two, (D) three AAA tracts. "n" denotes any nucleotide, but not A if adjacent to A. Same as in Figure S18, except profiled across the indicated A-rich motifs. Bin size: 1 nt.

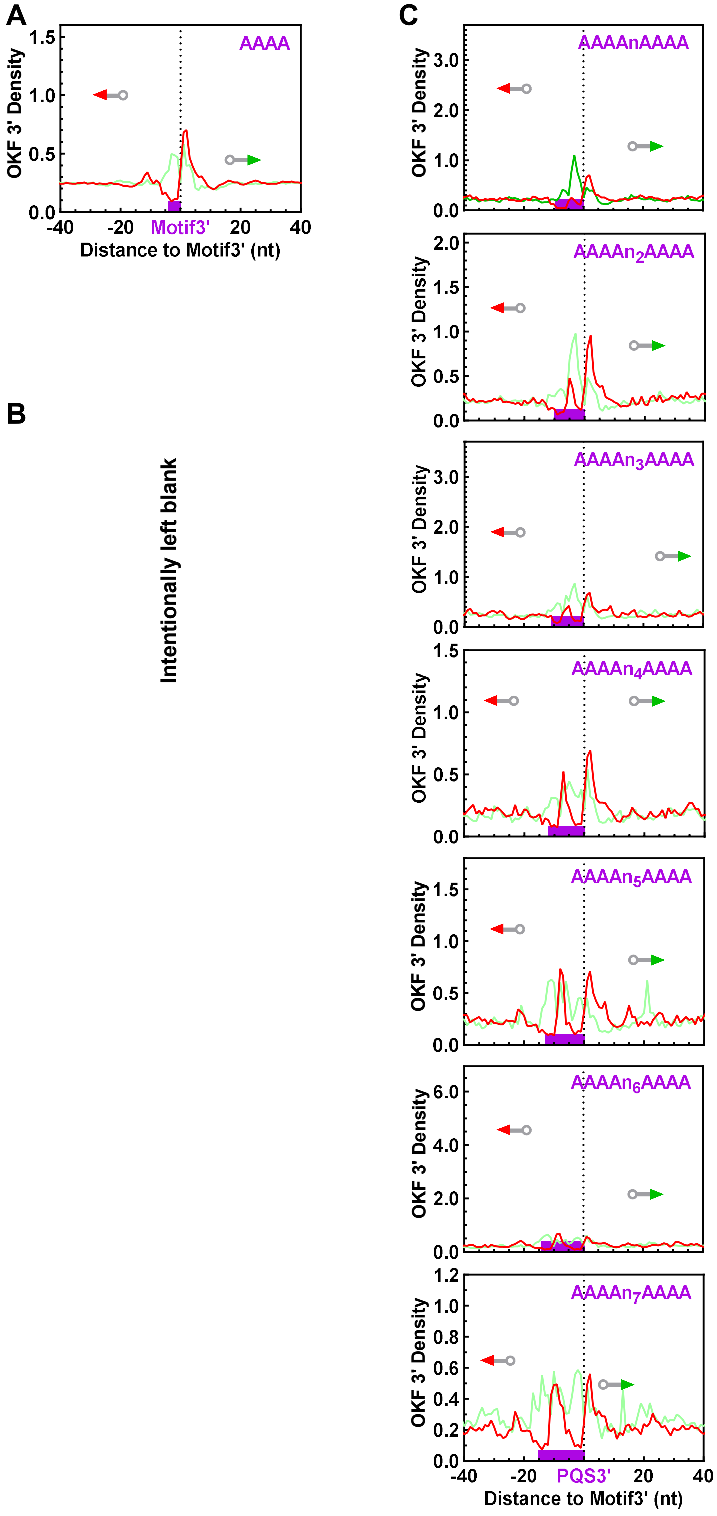

Figure S26. Distribution of OKF 3' ends at the 3' end of A-rich motifs containing (A) one and (C) two AAAA tracts. "n" denotes any nucleotide, but not A if adjacent to A. Same as in Figure S19, but profiled across the indicated A-rich motifs. Bin size: 1 nt.

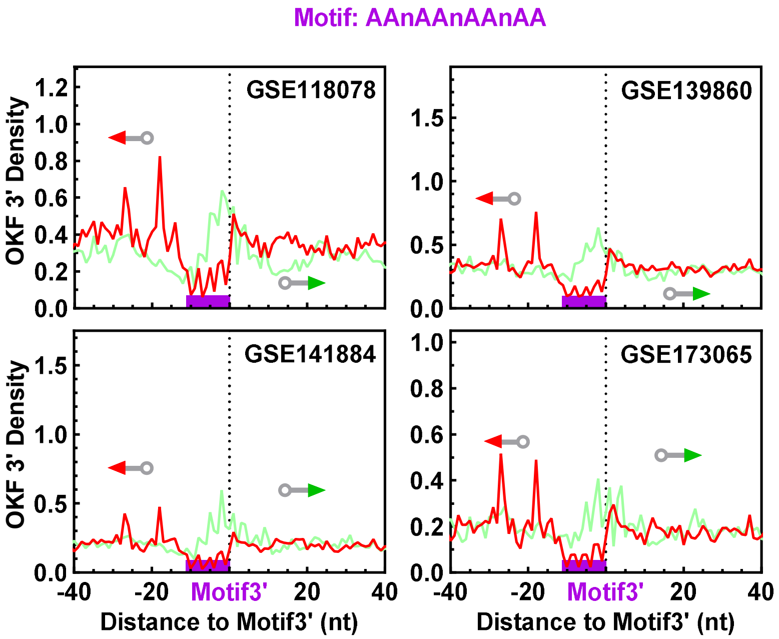

Figure S27. Distribution of OKF 3' ends at the 3' end of A-rich motifs with four AA tracts and 1-nt loops. "n" denotes any nucleotide other than A. Same as in Figure 10, except profiled across the indicated A-rich motifs. Bin size: 1 nt.

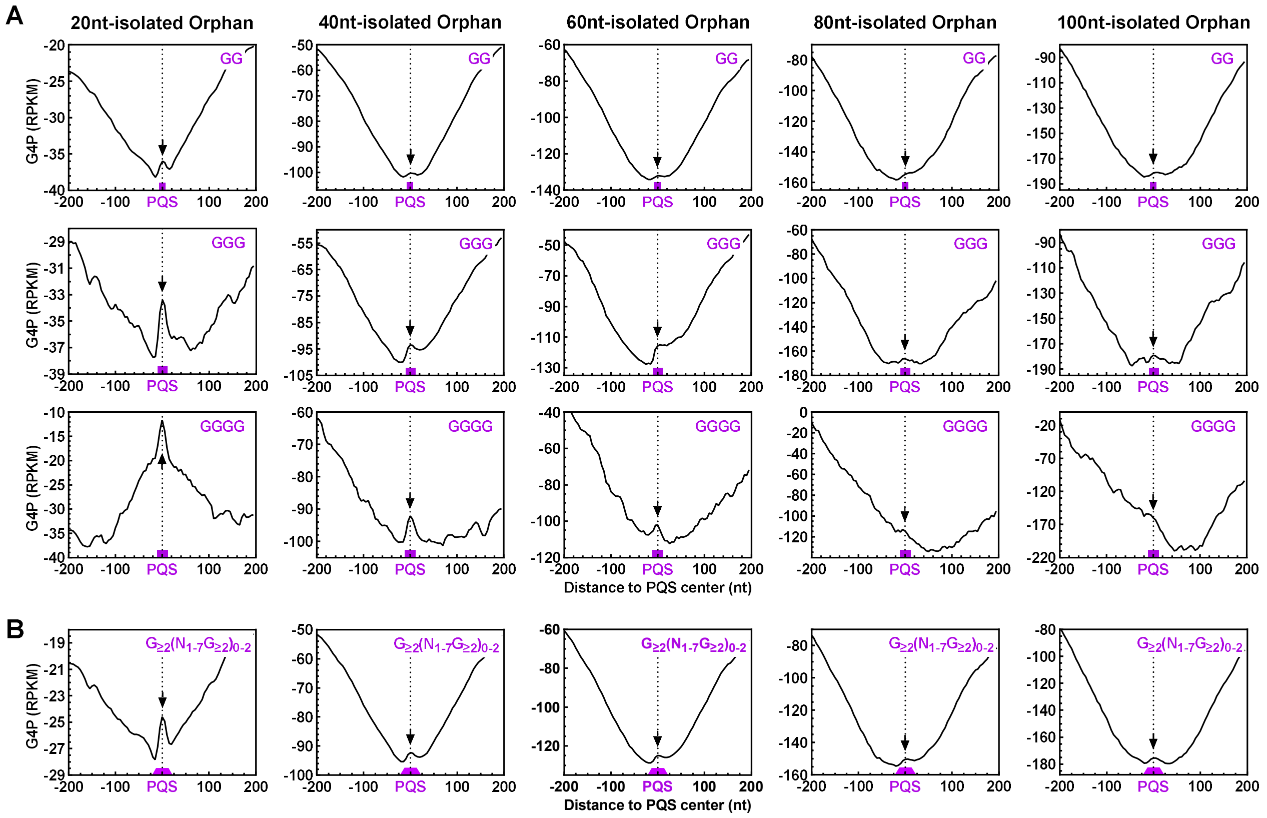

Figure S28. hG4 formation at orphan PQSs isolated from neighboring G-tracts by different numbers of nucleotides, detected by G4P enrichment at (A) single GG, GGG, GGGG tracts or (B) 1-3 $G_{\geq2}$ tracts. Bin size: 5 nt.

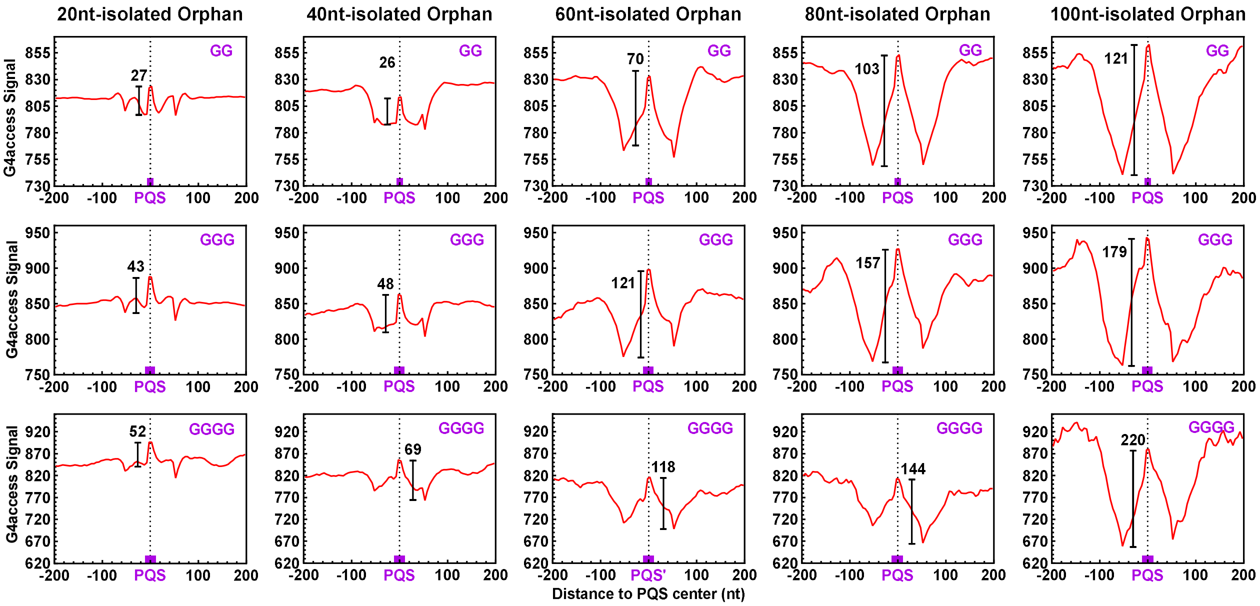

Figure S29. hG4 formation at orphan PQSs isolated from neighboring G-tracts by different numbers of nucleotides, detected by G4access at single GG, GGG, GGGG tracts. Bin size: 5 nt.

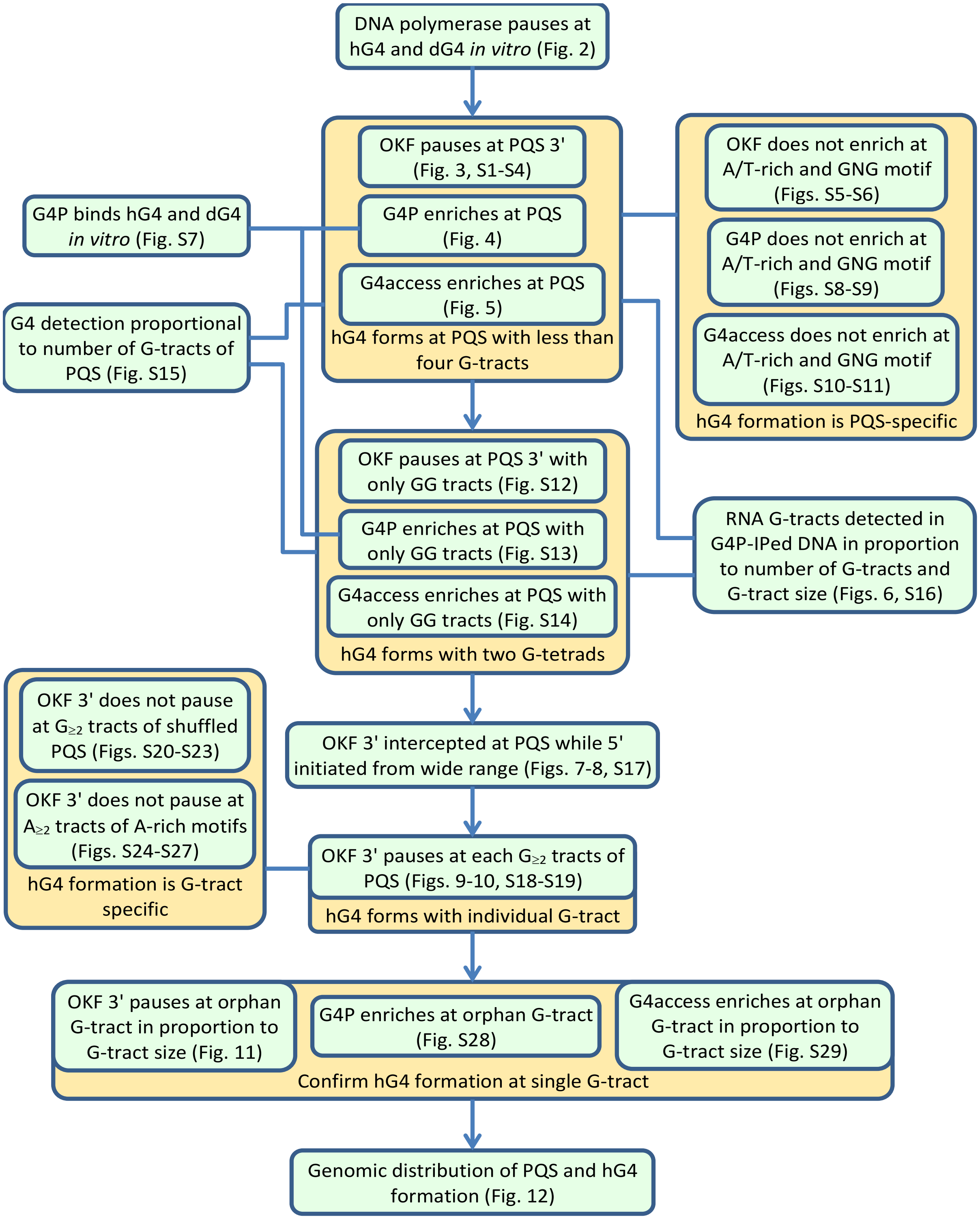

Figure S30. Summary of evidence and supporting information for the formation of hG4s in the genome of living yeast cells.

SI References

1. Zhang J-y, Zheng K-w, Xiao S, Hao Y-h, Tan Z (2014) Mechanism and Manipulation of DNA:RNA Hybrid G-Quadruplex Formation in Transcription of G-Rich DNA. *J. Am. Chem. Soc.* 136:1381-1390.
2. Gordenin DA, Porcella SY, Koussa NC, Tang CP, Kramer DN*, et al.* (2020) Separable, Ctf4-mediated recruitment of DNA Polymerase α for initiation of DNA synthesis at replication origins and lagging-strand priming during replication elongation. *PLoS Genet.* 16:e1008755.
3. Koussa NC, Smith DJ (2021) Post-replicative nick translation occurs on the lagging strand during prolonged depletion of DNA ligase I in Saccharomyces cerevisiae. *G3 Genes|Genomes|Genetics* 11:10.1093/g1093journal/jkab1205.
4. Gordenin DA, Koussa NC, Smith DJ (2021) Limiting DNA polymerase delta alters replication dynamics and leads to a dependence on checkpoint activation and recombination-mediated DNA repair. *PLoS Genet.* 17:e1009322.
5. Yeung R, Smith DJ (2020) Determinants of Replication-Fork Pausing at tRNA Genes in Saccharomyces cerevisiae. *Genetics* 214:825-838.
6. Kahli M, Osmundson JS, Yeung R, Smith DJ (2019) Processing of eukaryotic Okazaki fragments by redundant nucleases can be uncoupled from ongoing DNA replicationin vivo. *Nucleic Acids Res.* 47:1814-1822.
7. Xiao S, Zhang JY, Zheng KW, Hao YH, Tan Z (2013) Bioinformatic analysis reveals an evolutional selection for DNA:RNA hybrid G-quadruplex structures as putative transcription regulatory elements in warm-blooded animals. *Nucleic Acids Res.* 41:10379-10390.
8. Quinlan AR (2014) BEDTools: The Swiss-Army Tool for Genome Feature Analysis. *Curr Protoc Bioinformatics* 47:11 12 11-34.
9. Lubliner S, Keren L, Segal E (2013) Sequence features of yeast and human core promoters that are predictive of maximal promoter activity. *Nucleic Acids Res.* 41:5569-5581.
10. Lin Z, Wu W-S, Liang H, Woo Y, Li W-H (2010) The spatial distribution of cis regulatory elements in yeast promoters and its implications for transcriptional regulation. *BMC Genomics* 11:581.
11. Ramírez F, Dündar F, Diehl S, Grüning BA, Manke T (2014) deepTools: a flexible platform for exploring deep-sequencing data. *Nucleic Acids Res.* 42:W187-W191.
12. Zheng KW, Zhang JY, He YD, Gong JY, Wen CJ*, et al.* (2020) Detection of genomic G-quadruplexes in living cells using a small artificial protein. *Nucleic Acids Res.* 48:11706-11720.
13. Gietz RD, Schiestl RH, Willems AR, Woods RA (2004) Studies on the transformation of intact yeast cells by the LiAc/SS - DNA/PEG procedure. *Yeast* 11:355-360.
14. Cam HP, Whitehall S (2016) Chromatin Immunoprecipitation (ChIP) in Schizosaccharomyces pombe. *Cold Spring Harb. Protoc.* 2016.
15. Zheng K-w, Xiao S, Liu J-q, Zhang J-y, Hao Y-h*, et al.* (2013) Co-transcriptional formation of DNA:RNA hybrid G-quadruplex and potential function as constitutional cis element for transcription control. *Nucleic Acids Res.* 41:5533-5541.
16. Esnault C, Magat T, Zine El Aabidine A, Garcia-Oliver E, Cucchiarini A*, et al.* (2023) G4access identifies G-quadruplexes and their associations with open chromatin and imprinting control regions. *Nat. Genet.* 55:1359-1369.
17. Langmead B, Salzberg SL (2012) Fast gapped-read alignment with Bowtie 2. *Nat. Methods* 9:357-359.
18. Li H, Handsaker B, Wysoker A, Fennell T, Ruan J*, et al.* (2009) The Sequence Alignment/Map format and SAMtools. *Bioinformatics* 25:2078-2079.
